# A supervised data-driven spatial filter denoising method for speech artifacts in intracranial electrophysiological recordings

**DOI:** 10.1162/imag_a_00301

**Published:** 2024-10-01

**Authors:** Victoria Peterson, Matteo Vissani, Shiyu Luo, Qinwan Rabbani, Nathan E. Crone, Alan Bush, R. Mark Richardson

**Affiliations:** Department of Neurosurgery, Massachusetts General Hospital, Harvard Medical School, Boston, MA, United States; Instituto de Matemática Aplicada del Litoral, IMAL, UNL, CONICET, Santa Fe, Argentina; Department of Biomedical Engineering, The Johns Hopkins University School of Medicine, Baltimore, MD, United States; Department of Electrical & Computer Engineering, The Johns Hopkins University, Baltimore, MD, United States; Department of Neurology, The Johns Hopkins University School of Medicine, Baltimore, MD, United States; Department of Brain and Cognitive Sciences, Massachusetts Institute of Technology, Cambridge, MA, United States

**Keywords:** speech production, speech artifact, iEEG, spatial filtering, phase-coupling optimization

## Abstract

Neurosurgical procedures that enable direct brain recordings in awake patients offer unique opportunities to explore the neurophysiology of human speech. The scarcity of these opportunities and the altruism of participating patients compel us to apply the highest rigor to signal analysis. Intracranial electroencephalography (iEEG) signals recorded during overt speech can contain a speech artifact that tracks the fundamental frequency (F0) of the participant’s voice, involving the same high-gamma frequencies that are modulated during speech production and perception. To address this artifact, we developed a spatial-filtering approach to identify and remove acoustic-induced contaminations of the recorded signal. We found that traditional reference schemes jeopardized signal quality, whereas our data-driven method denoised the recordings while preserving underlying neural activity.

## 1 Introduction

Human intracranial recordings, that is, in-vivo electrophysiological signals acquired during specific neurosurgical treatments such as focal epilepsy and deep brain stimulation (DBS), have enabled the study of neural responses with high temporal and spatial resolution, in both surface and deep structures of the human brain during behavioral tasks (Tekriwal et al., 2018). The study of speech motor control especially benefits from awake intraoperative recordings during which local field potentials (LFP) and single unit activity of subcortical targets can be simultaneously acquired during speech production (Lipski et al., 2018;Ma et al., 2022).

Intracranial EEG (iEEG) provides much better spatial specificity and higher signal-to-noise-ratio (SNR) than surface EEG. However, iEEG can also suffer from eye movement, muscle, and even cardiac artifacts (Mercier et al., 2022). To develop invasive brain–computer interfaces (BCI) for speech prostheses, the neural activity in the high-gamma band (60 – 200 Hz) is typically used for speech decoders (Angrick et al., 2019;Anumanchipalli et al., 2019;Moses et al., 2021;Proix et al., 2022). Recently, we and others have shown that electrocorticography recordings may contain artifacts associated with the mechanical vibrations produced by the participant’s voice or sounds played by a loudspeaker (Bush et al., 2022;Roussel et al., 2020). Although not always present, the acoustic-induced artifact can appear in different setups, recording modalities (e.g., ECoG, µECoG, and Utah arrays), and experimental conditions, as shown by Roussel et al. who detected the artifact in data from three out of five centers analyzed (Roussel et al., 2020). We have also detected the artifact in local field potentials recorded from DBS leads or stereotactic mapping electrodes (Bush et al., 2022). Interestingly, the artifact has not yet been observed in stereo EEG data (sEEG). For overt speech experiments, this speech artifact shares spectral characteristics with the produced audio signal, being locked at the fundamental frequency (F0) of the participant’s voice (Bush et al., 2022). The overlap between typical human F0 (between 70 and 240 Hz) and high-gamma activity (60 to 250 Hz) imposes the need to account for this speech artifact to study the brain activity associated with speech production.

**Fig. 1.**
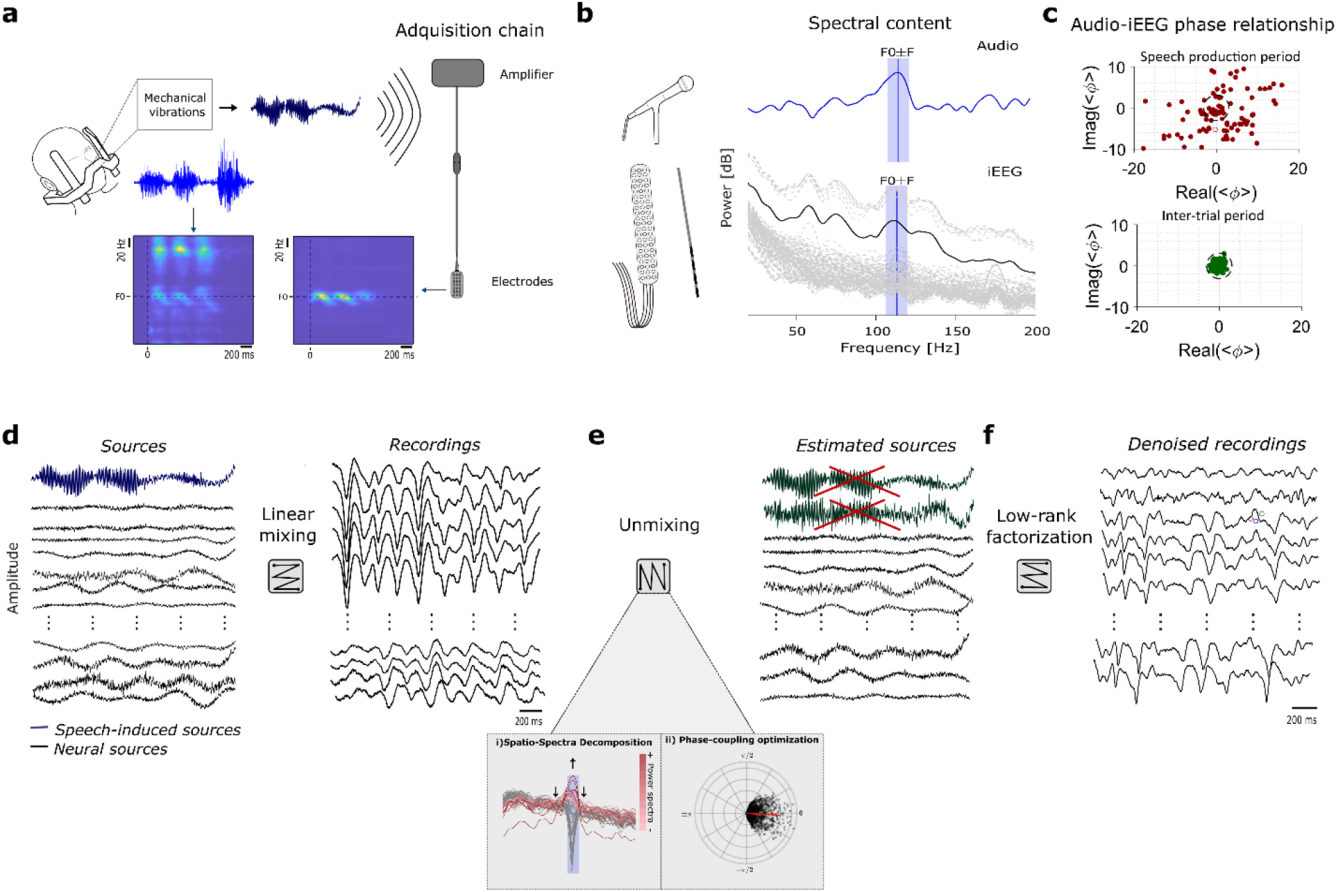
Speech-induced speech artifact model assumptions. (a) Schematic representation of how speech-induced mechanical vibrations can affect neural recordings during stereotactic frame surgery. The frame attached to the patient’s head acts as a resonance system with the skull, transmitting and distorting speech-induced vibrations that can affect the neural recordings’ acquisition chain. An artifactual electrode has a suspicious narrow-band component that resembles the audio time–frequency information, as illustrated in the average spectrogram plots calculated across speech-produced epochs. (b) The speech artifact tracks the fundamental frequency (F0) of the participant’s voice. (c) The coherence between the audio signal and the neural recordings across trials (<ϕ>) can assess the level of speech artifact contamination in each electrode. During speech production, many channels present artifact (lie outside the dashed circumference). (d) The recordings, at the amplifier level, can be thought as a linear mixing between the speech artifact and brain-related sources. (e) The proposed unmixing pipeline is based on two spatial-filtering methods. While the Spatio-Spectra Decomposition (SSD) helps to enhance the signal-to-noise ratio in the frequency band of interest, the phase-coupling optimization seeks at finding the sources with the highest coherence value with respect to the audio. (f) By means of low-rank factorization, the signal reconstruction is done zeroing out the speech artifactual sources.

As shown before, the acoustic artifact is introduced along the acquisition chain, where the mechanical vibrations of the acoustic signal are translated into voltage (Roussel et al., 2020). Passive electrical components can exhibit an electrical response when stressed physically, a phenomenon referred to as the*microphonic effect*(Nelson & Davidson, 2002). This effect can be exacerbated in the case of speech tasks performed during stereotactic neurosurgery, at which the patient’s head is fixed to a stereotactic frame (Fig. 1a). This frame may act as a resonance system that exacerbates speech-induced vibrations originating in the larynx and traveling through the head and skull. Speech-induced vibrations, which look like a distorted version of the speech audio, can affect the electrodes and acquisition chain. In affected electrodes, the time–frequency representation shows narrow-band components that resemble the audio spectrogram rather than true neural activity, as shown inFigure 1a. This narrow-band component tracks the fundamental frequency of the participant’s voice (Fig. 1b). Acoustic-induced vibration artifacts can be detected by measuring the coherence value between the speech acoustic signal and neural recordings in the high-gamma frequency band (Bush et al., 2022) (Fig. 1c). This coherence value varies largely between and within patients, with no clear spatial pattern across recording channels (Bush et al., 2022). This indicates that the speech artifact is a channel-specific type of noise, and thus traditional re-reference schemes may jeopardize the quality of the neural recordings (Liu et al., 2015).

Considering the recorded brain activity as superpositions of different statistical sources (Sabbagh et al., 2020), the brain signals at the amplifier level can be thought of as the consequence of a linear mix between true neurophysiological and non-neural sources, including speech-induced vibrations (Fig. 1d). Using spatial filter methods, these sources can be untangled and estimated from the multivariate (multichannel) electrode signals (Schaworonkow & Voytek, 2021). As such, traditional re-referencing schemes used in neurophysiology, such as bipolar, Laplacian, or common average reference (CAR), can be reframed as spatial-filtering approaches, in which the recorded brain signals are multiplied by a predefined matrix that references the recording of one electrode with respect to a neighborhood of channels (Cohen, 2022) (seeSection 2.5.1).

Data-driven spatial filters offer a more flexible re-reference scheme than traditional methods. They can be used for denoising, using linear transformations to estimate the data subspace related to “noise” and discard it for the subsequent signal analyses (Cohen, 2017,^2022^;Haufe et al., 2014). This approach has been used primarily for noninvasive electrophysiology (Cohen, 2017,^2022^;Haufe et al., 2014;Wallstrom et al., 2004), and more recently for iEEG signal processing (Mercier et al., 2022;Michelmann et al., 2018;Schaworonkow & Voytek, 2021). A typical pipeline for artifact removal in noninvasive EEG consists of using principal component analysis (PCA) and independent component analysis (ICA) for identifying artifact components (Jung et al., 2000;Wallstrom et al., 2004). PCA is commonly used as a dimensionality reduction tool to alleviate the decomposition done by ICA. Then, by means of low-rank factorization, backward and forward projections are made between the signal and the source space, identifying and discarding those components related to artifacts.

For the case of speech-related EMG artifacts (originating by movement of articulators), ICA-based methods were postulated as promising denoising approaches for muscular speech-induced artifact removal in surface EEG for event-related potential analyses, which do not investigate high-gamma activity (Porcaro et al., 2015;Vos et al., 2010). A key difference between muscular and vibration-induced speech artifacts is that while both overlap with high-gamma activity, the narrow-band characteristic of the vibration speech artifact may lead to poor ICA-based denoising performance due to its limitations in decomposing mixed narrow-band oscillations (Nikulin et al., 2011) (see alsoSection 3).

Here, we introduce phase-coupling decomposition (PCD), an algorithm for acoustic-induced artifact rejection. This algorithm performs data-driven spatial-filtering denoising based on low-rank factorization. It is designed to separate acoustic-induced artifactual sources from neural sources via a phase-coupling decomposition. The spatiospectral decomposition (SSD) (Nikulin et al., 2011) algorithm is used first to enhance signal-to-noise ratio around F0 and perform dimensionality reduction to help the search of artifactual components (Fig. 1e). The phase-coupling optimization (PCO) (Waterstraat et al., 2017) method is then applied to identify sources phase locked to the acoustic signal (Fig. 1e). Thus, the coherence between the audio and the neural data is optimized, allowing retrieval of those sources related to acoustic-induced noise. Similar to the ICA-based artifact pipeline mentioned above, signal reconstruction is based on low-rank factorization, discarding the detected artifactual components (Fig. 1f).

**Fig. 2.**
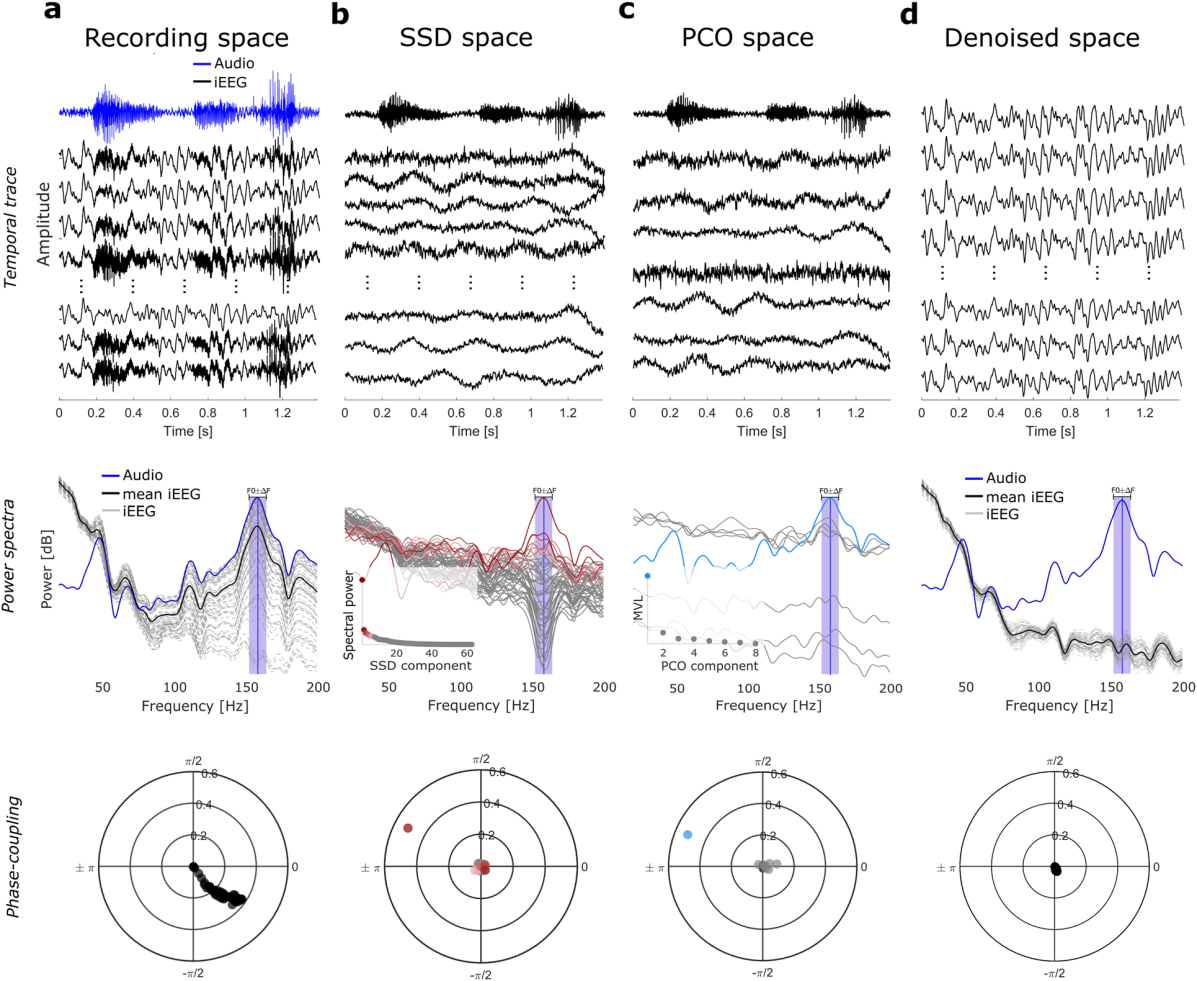
Illustration of the phase-coupling decomposition pipeline. Space transformations from the recording space to the clean space in the temporal, power, and phase domains. (a) (Recording space), Top: iEEG recordings (black traces) were combined with the recorded audio (blue trace) to simulate the contaminated recordings for illustration purposes. Middle: note the similarity of audio and iEEG power spectra. Bottom: Polar plot showing phase-coupling value between the audio and iEEG channels (represented as dots). (b) (SSD space) SSD identifies components that maximize power around F0 (color coded in the spectral domain from dark red to light pink). (c) (PCO space) Only those SSD components with the strongest power around F0 are used to compute PCO. The MVL is optimized and only those PCO components that show the highest coherence with the audio are identified as artifactual (light-blue trace). Here, the first eight components are shown. (d) (Denoised space) via low-rank factorization, the artifactual component(s) are excluded for signal reconstruction. Note the clean iEEG traces, with no peak around F0 in the power spectra and phase-coupling values centered at zero.

**Fig. 3.**
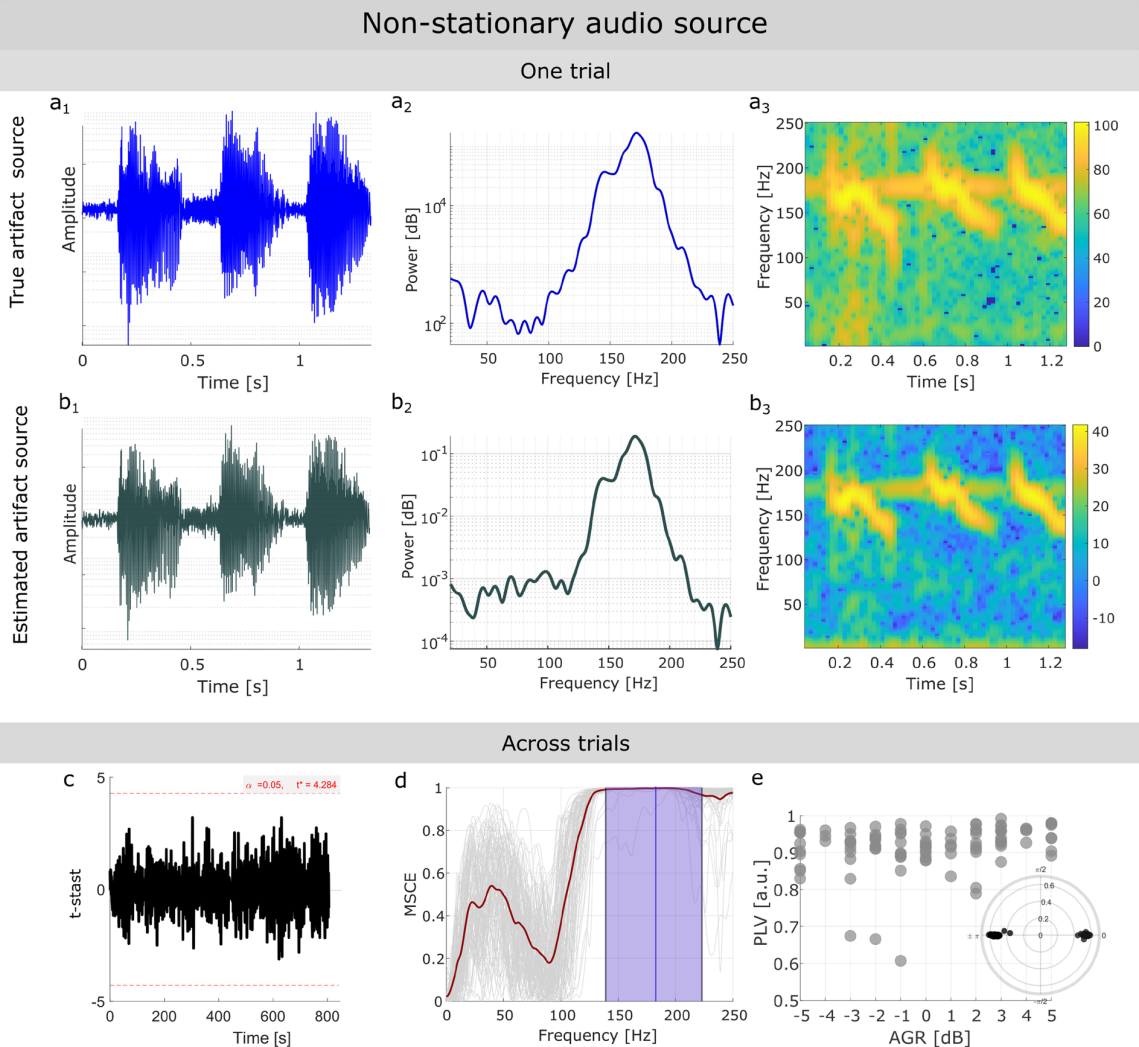
PCD correctly retrieves nonstationary and weak artifactual sources. Representative performance of PCD on an artifactual source with a nonstationary signal profile and an artifact-to-physiological gamma ratio (AGR) = 2dB. (Top panel) (a1-a3), Visualization in the time, frequency, and time–frequency domain of the ground-truth artifactual source for a given trial. (b1-b3), Same as above for the retrieved artifactual source for the same trial. (Bottom panel) PCD performance across trials. (c) One-dimensional statistical parametric mapping was used to evaluate statistical similarities at each sample point between the true and estimated source for each trial. No significant differences (t-stats values < t*) were found at any time point. (d) Magnitude-squared coherence estimate (MSCE) between true and estimated artifact sources. Gray lines represent individual trials. Mean MSCE across trials is denoted by the dark red line. The mean F0 across trials is shown as the blue vertical line, while the violet band indicates the SAFB. The mean MSCE value in the SAFB was always above 0.97, regardless of the variable AGR across trials. (e) Phase-locking value (PLV) across simulated trials at different AGR between the true and estimated artifact source, for each trial. Inset on the right corner aggregates the phase difference found across trials between the true and estimated artifact source.

**Fig. 4.**
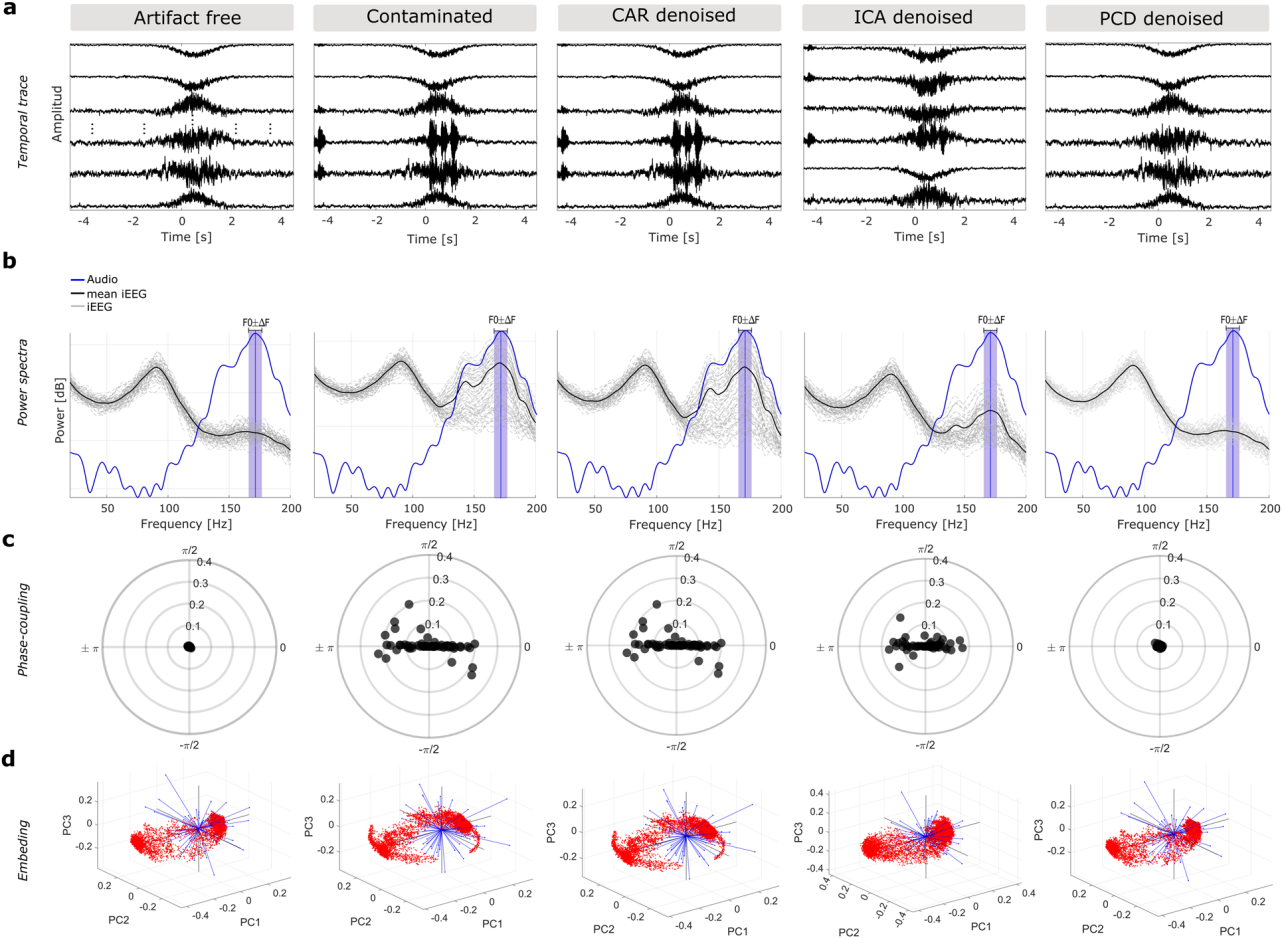
PCD removes acoustic-induced speech artifact while preserving neural activity in simulated data, outperforming previous methods. Impact on signal quality of applying CAR, ICA, and PCD for a trial of the realistic simulation (low-amplitude broadband gamma modulation, AGR = -2 dB and nonstationary artifact source). Temporal trace (a), power spectra (b), and phase-coupling plots (c) as inFigure 2. (a) Resulting temporal traces with time reference relative to the speech onset (t = 0). (b) Power spectrum of each resulting iEEG signal (gray lines) and the recorded audio (blue line). The mean power spectrum across iEEG channels is shown by the thick black line. Violet shades indicate the SAFB. (c) The phase relationship between the audio and the resulting brain signals. Each dot represents a channel. (d) Biplots of the PC subspace described by the first three principal components (PC1, PC2, PC3). Red dots represent scores while blue lines represent the loading directions. The shape of the red cloud illustrates the PCA embedding.

**Fig. 5.**
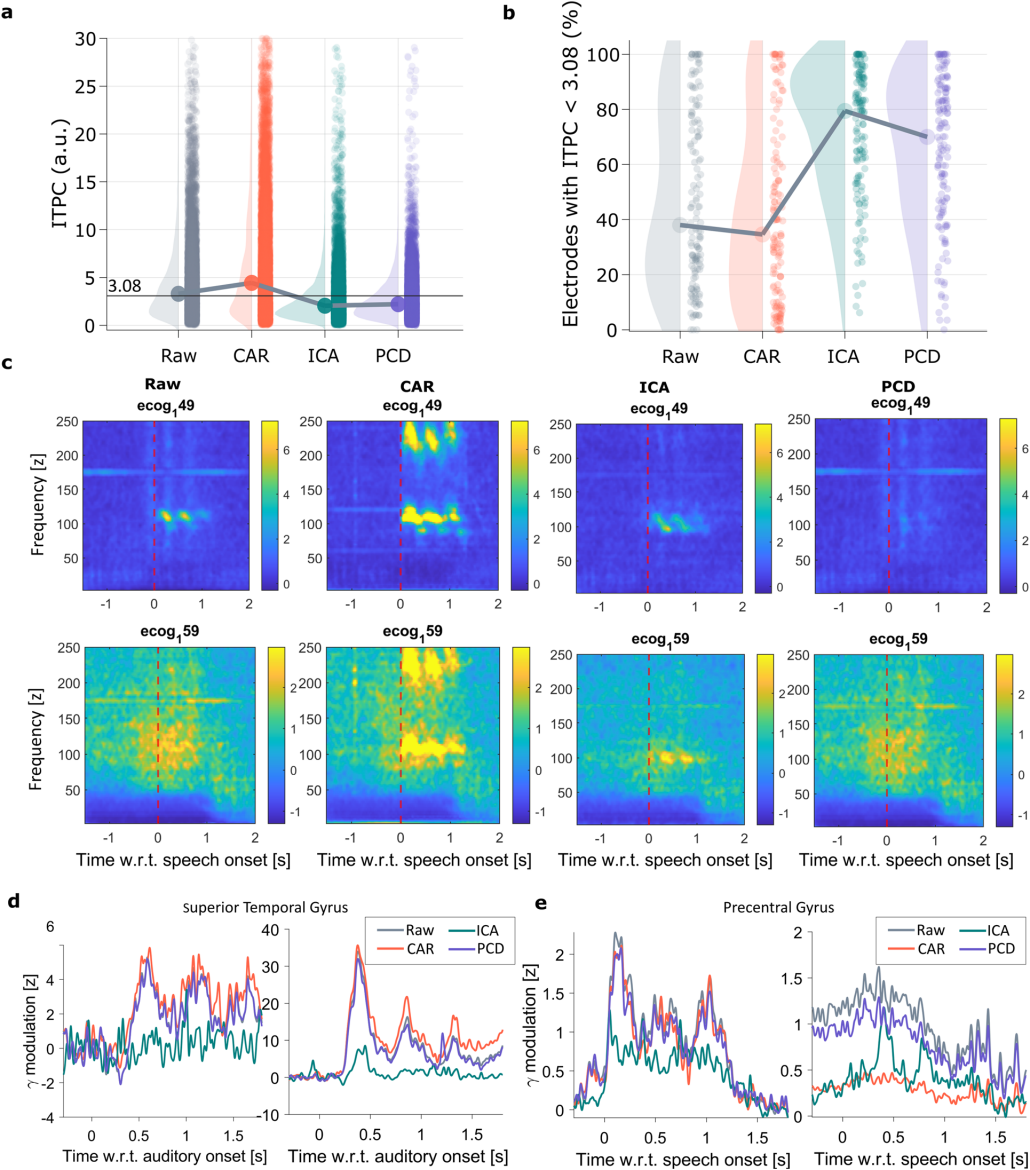
PCD removes the speech artifact while preserving underlying γ-activity in intracranial recordings. (a) Coherence value between a channel and the recorded audio, measured by means of ITPC. Here a dot represents a channel, and the horizontal solid line indicates the significance ITPC threshold (3.08). (b) Percentage of electrodes that lie below the significant ITPC threshold, where every dot represents the data from a recording session. (c) Time–frequency plots for raw signals and effect of applying each denoising method in an electrode with strong speech artifact (top row) and an electrode with physiological speech-related γ-activity (bottom row). Time is relative to speech onset. (d) Gamma profiles (z-scored normalized) during stimulus onset of two electrodes located in the superior temporal gyrus for two different patient’s data. (e) Gamma profiles (z-scored normalized) during speech onset of two electrodes located in the precentral gyrus for two different patient’s data. Different color traces are used for the raw and the resulting denoised data via each tested method.

**Fig. 6.**
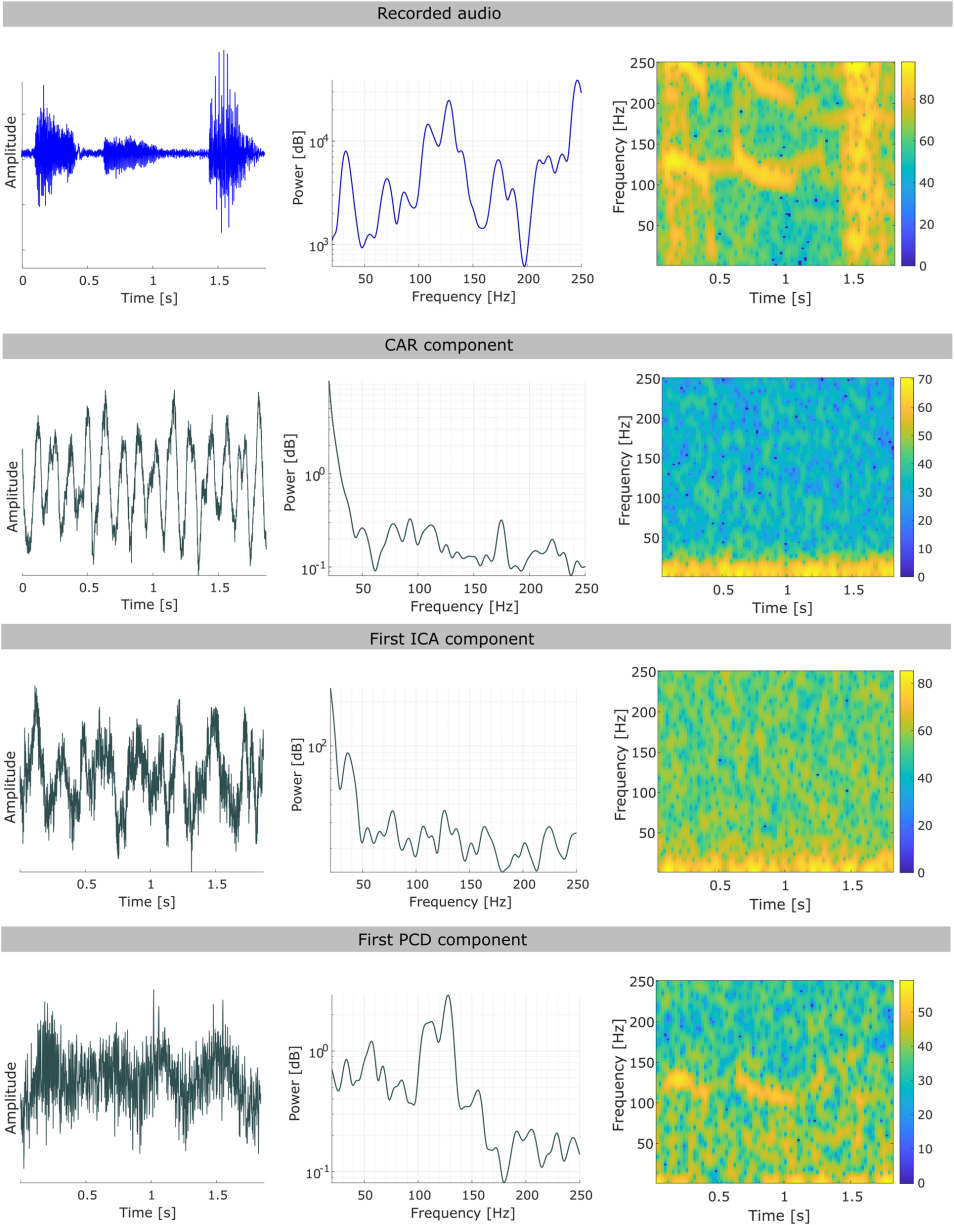
Artifact retrieval comparison. Comparison in the temporal, frequency, and time–frequency domain of the recorded audio and artifactual component estimated by CAR, ICA, and PCD. For a given participant, at a randomly selected trial, the component with the highest phase relationship with the audio is shown for ICA and PCD, while for CAR, average across trials is depicted.

**Fig. 7.**
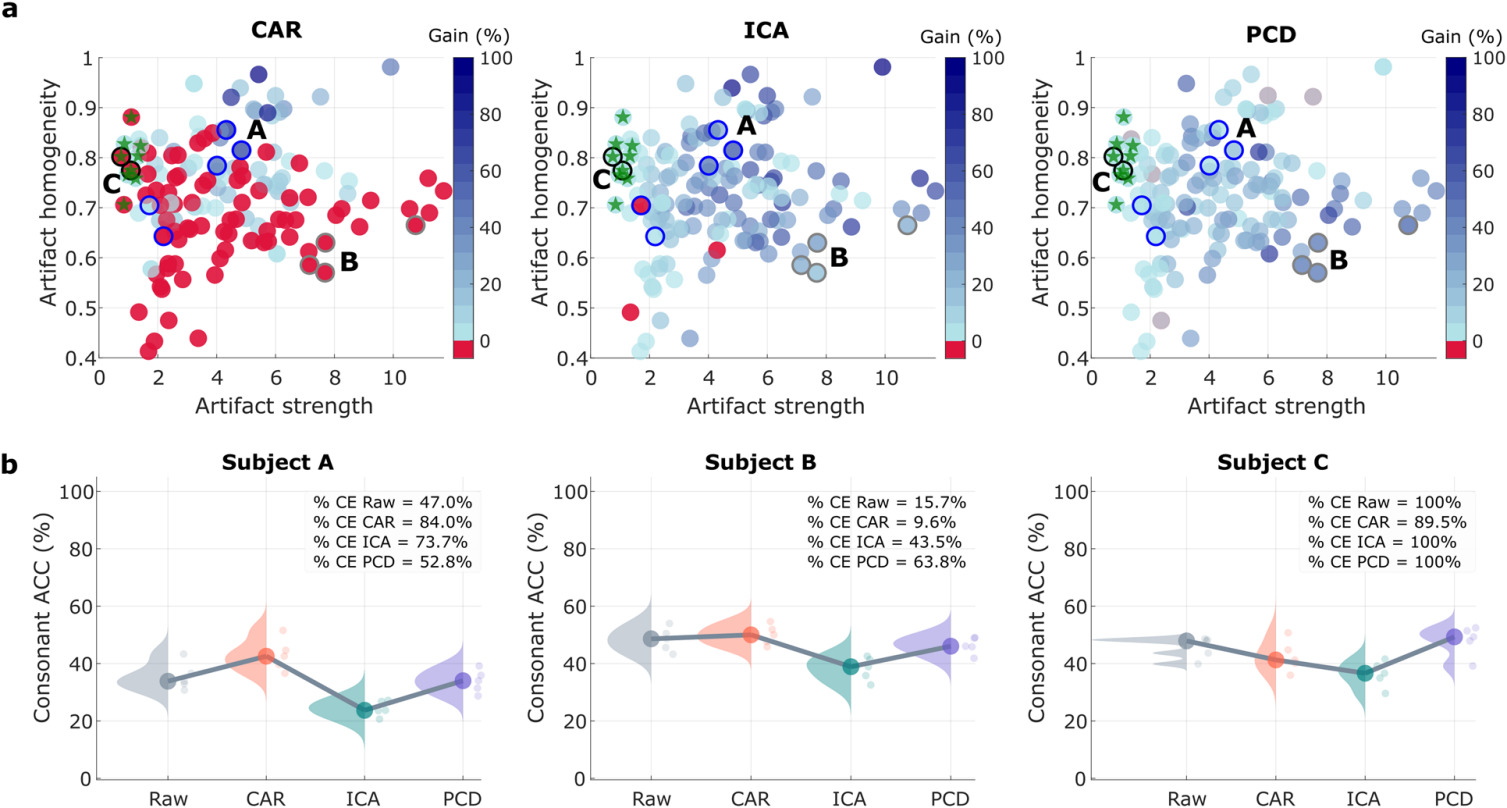
Understanding the impact of each denoising method as a preprocessing step. (a) Percentage gain in electrodes below the significant ITCP threshold (Gain) versus artifact homogeneity and strength, for CAR, ICA, and PCD. Each dot represents the data from a recording session. Raw data with no detectable speech artifact are marked with a green star. Data from three subjects (A, B, and C) are highlighted: Homogeneous artifact case (Subject A); strong artifact case (Subject B), and no artifact case (Subject C) represented as circles with blue, gray, and black contours, respectively. (b), Impact in speech decoding from neural data (detection of consonant–vowel syllables) in three cases: (i) clean electrode (CE) gain is higher for CAR and ICA than for PCD (Subject A), (ii) CE gain is better with PCD than for the other two methods (Subject B), and (iii) data without speech artifact (Subject C). ACC stands for accuracy, that is, the number of correct classified samples over the total of samples. Every point represents the accuracy of the testing data on a 5-fold cross-validation scenario.

First, we demonstrate how PCD cleans acoustic-induced artifacts from an affected recording (Fig. 2). Then, we test the denoising performance of this algorithm in simulated data, in which the artifact and the neural sources are artificially generated and/or mixed. The algorithm successfully recovers the artifactual source, in the time, frequency, and phase domains even when dealing with highly nonstationary audio signals and high SRN (Fig. 3). Importantly, we demonstrate the algorithm’s ability to denoise while preserving neural data and compare its performance with respect to traditional spatial-filtering methods such as CAR and ICA (Fig. 4). Finally, we test the PCD denoising algorithm in real acoustic contaminated iEEG data, showing a significant reduction of the extent and number of artifact-affected electrodes while preserving the underlying speech-related neurophysiological response (Figs. 5–7).

## 2 Materials and Methods

### 2.1 The phase-coupling decomposition algorithm

We consider the brain recordings as a linear combination of statistical sources, where at least one of those sources is related to the speech artifact, which can be detected by measuring the coherence value between the audio signal and the neural recordings (Bush et al., 2022). The recorded brain signals are considered then as terms of a linear forward model, in which$N_{C}$statistical sources are projected into$N_{C}$channels along the$N_{S}$data samples via a linear mixing process:

X=AS, (1)

where$\text{X}\in {\mathbb{R}}^{{\text{N}}_{\text{c}}\times {\text{N}}_{\text{s}}}$denotes the recorded brain signals,$\text{S}\in {\mathbb{R}}^{{\text{N}}_{\text{c}}\times {\text{N}}_{\text{s}}}$is the source activity, and$\text{A}\in {\mathbb{R}}^{{\text{N}}_{\text{c}}\times {\text{N}}_{c}}$denotes the mixing matrix. Each columnaof the mixing matrixAis known as*spatial pattern*and describes the projection of an individual source to the sensor space.

The idea of PCD is to find the artifactual sources that are phase coupled with the acoustic signal, denoted here as$z\in {\mathbb{R}}^{{\text{N}}_{\text{s}}}$. In that respect, PCD seeks to find the artifactual sources by solving the inverse problem related to (1) so that:

$$\hat{\text{S}}={\text{W}}_{PCD}^{T}\text{X},$$ (2)

where T denotes transpose and$\hat{\text{S}}\in R^{N_{c}\times N_{s}}$is a matrix of whose columns have the estimated artifactual sources.

The denoising pipeline can be thought of as a supervised two-step spatial-filtering procedure which performs data cleaning based on low-rank factorization (Haufe et al., 2014). As shown inBox 1, the speech artifact rejection comprises four main steps: (1) band-width estimation, (2) spatiospectral decomposition, (3) phase-coupling optimization, and (4) signal reconstruction. In the following subsections, each step is described.

Box 1. Phase-Coupling Decomposition

Given the recorded brain activity$\text{X}\in {\mathbb{R}}^{{\text{N}}_{\text{c}}\times {\text{N}}_{\text{s}}}$, where$N_{c}$and$N_{s}$are the number of channels and samples, respectively, and an acoustic signal$\text{z}\in {\mathbb{R}}^{{\text{N}}_{\text{s}}}$, this algorithm identifies narrow-band components ofXthat are phase coupled withz.

**Step 1:**Estimate the speech artifact bandwidth.

1. Calculate the power spectrum of*z*.
2. Around the fundamental frequency (F0) of the voice, find the peak in the spectra ($P_{F0}$).
3. Define the center$P_{F0}$and bandwidth of the speech artifact frequency band (SAFB):$F_{c}\pm \;\Delta \;F_{c}$.

**Step 2:**Find projections ofXthat maximize the signal-to-noise ratio (SNR) around$F_{c}$via the spatiospectral decomposition (SSD) (Nikulin et al., 2011).

1. Define as*signal band*the SAFB:$F_{c}\pm \;\Delta \;F_{c}$, and as*noise band all frequencies*except SAFB. Find the projection matrix${\text{W}}_{SSD}$by solvingequation (3)and project the data on the firstkSSD components${\tilde{\text{W}}}_{SSD}=\left[{\text{w}}_{1},\ldots ,{\text{w}}_{\text{k}}\right]$, as follows:${\tilde{\text{X}}}_{SSD}={\tilde{\text{W}}}_{SSD}^{T}\text{X}$.

**Step 3**: Find the artifactual sources via the phase-coupling optimization algorithm (PCO) (Waterstraat et al., 2017).

1. In the SSD space, find the projection matrix${\text{W}}_{PCO}$which maximizes the mean vector length (seeequations (6)and(7)) between${\tilde{\text{X}}}_{SSD}$andz**.**
2. Compute the PCO components${\text{X}}_{PCO}={\text{W}}_{PCO}^{T}{\text{X}}_{SSD}$and identify themacoustic-induced sources.

**Step 4:**Perform the denoising via low-rank factorization.

1. Compute the PCD unmixing matrix${\text{W}}_{PCD}$which projectsXto the artifact source space. This matrix is the conjunction of both${\text{W}}_{PCO}$and${\text{W}}_{SSD}$(equation (8)).
2. Compute the PCD mixing matrix via the pseudoinverse:

${\text{A}}_{PCD}={\text{W}}_{PCD}^{†}$

3. Reconstruct the signal by using ($N_{c}-m$) components:${\text{X}}_{denoised}={\tilde{\text{A}}}_{PCD}{\tilde{\text{W}}}_{PCD}^{T}\text{X}$.

#### 2.1.1 Speech artifact band-width estimation

The speech artifact is a narrow-band component noise. It happens around the fundamental frequency (F0) of the participant voice (Bush et al., 2022). The recorded audio signalzis used as a proxy for the speech artifact frequency band estimation. We use Welch’s method (Welch, 1967) for calculating the power spectra of the audio (detrended signal). An estimate of F0 from audio recordings is used as a starting point to find a peak in gamma band (50–250 Hz). Here, the Gaussian curve fitting algorithm is used to calculate the central frequency ($F_{C}$) and band width ($\Delta F_{c}$) of the speech artifact frequency band (SAFB) by estimating the mean and full width at half maximum of the resulted fitted signal. The SAFB is then defined as$F_{c}\pm \;\Delta F_{c}$.

#### 2.1.2 Spatiospectral decomposition

The spatiospectral decomposition algorithm (SSD) is a spatial-filtering approach that allows to maximize the signal power at a given frequency peak while minimizing it for surrounding frequencies (Nikulin et al., 2011). The method assumes that the recorded brain activity is a superposition of narrow-band oscillations at the given frequency peak of interest (*signal*) and neighboring frequency bands (*noise*), that is$\text{X}={\text{X}}_{signal}+{\text{X}}_{noise}$. By traditional temporal filters, such as the Butterworth zero-phase filter, the signal and noise contributions toXcan be separated (Nikulin et al., 2011).

The objective of SSD is to find a set of spatial filters (columns of an unmixing matrix${\text{W}}_{SSD}$) such that the power of the signal is maximized while the power of the noise is minimized:

$$\text{w}=argmax_{\text{w}\;\in \;\;{\mathbb{R}}^{N_{C}}}\frac{{\text{w}}^{T}\Sigma_{\text{signal}}\text{w}}{{\text{w}}^{T}\Sigma_{\text{noise}}\text{w}},$$ (3)

where$\Sigma_{\text{signal}}=\;{\text{X}}_{signal}^{T}{\text{X}}_{signal\;}$and$\Sigma_{\text{noise}}=\;{\text{X}}_{noise}^{T}{\text{X}}_{noise\;}$are the covariance matrices on the*signal band*and*noise band*, respectively.

The number of components returned by SSD is equal to the number of channels given inX, sorted according to the relative power spectrum in the frequency band of interest. Using the firstkSSD components, dimensionality reduction is achieved by projecting the data on the reduced SSD space as follows:

$${\tilde{\text{X}}}_{SSD}={\tilde{\text{W}}}_{SSD}^{T}\text{X},$$ (4)

where${\tilde{\text{W}}}_{SSD}=\left[{\text{w}}_{\text{SS}{\text{D}}_{1}},\ldots ,{\text{w}}_{\text{SS}{\text{D}}_{\text{k}}}\right].$

In the context of the vibration artifact denoising, we consider here that our frequency band of interest is the SAFB. Also, the data projected on the firstkSSD components${\tilde{\text{X}}}_{\text{SSD}}$are used as a good starting point to run the second spatial-filtering technique involved in the PCD pipeline.

#### 2.1.3 Phase-coupling optimization

Phase-coupling optimization (PCO) is a supervised spatial-filtering algorithm developed by Waterstraat et al. that seeks to recover neural sources that are phase coupled to an external target variable (Waterstraat et al., 2017). The optimization criterion is based on the maximization of the mean vector length (Canolty et al., 2006) (MVL), a differentiable metric that results in values different from zero when a phase coupling exists. Within this denoising pipeline, we take advantage of the PCO formulation and extend it in order to find the artifactual sources underlying neural activity. Phase coupling is then computed via the MVL between the SSD data projection${\tilde{\text{X}}}_{\text{SSD}}$and the best guess of the artifact source. Considering that data in SSD space belong to the complex domain, the analytic signal${\text{Y}}_{SSD}\in \mathbb{C}$should be first obtained. The Hilbert transform is utilized to find the imaginary part such that

$${\text{Y}}_{SSD}={\text{X}}_{SSD}+iℋ\left({\text{X}}_{SSD}\right)\in \mathbb{C},$$ (5)

whereidenotes the imaginary unit defined so as$i^{2}=-1$. Therefore, the MVL is defined by

$$MVL=\left|\frac{1}{N_{S}}\sum_{\text{t}=1}^{N_{S}}\frac{z_{t}{\text{w}}^{T}{\text{y}}_{t}}{\left|{\text{w}}^{T}{\text{y}}_{t}\right|}\right|\text{​}.$$ (6)

Note that phase coupling is calculated across sample points, such that MVL represents a summary of the phase relation across a given time window. Assuming that the artifact source is the recorded audio signalz, a linear filter${\text{w}}_{PCO}=\text{w}=\left[w_{1},\ldots ,w_{k}\right]$is found by maximizing the mean vector length at each sample point between the acoustic signal and the SSD components, as follows:

$${\text{w}}_{PCO}=argmax_{\text{w}\;\in \;{\mathbb{R}}^{k}}\left|\frac{1}{N_{S}}\sum_{\text{t}=1}^{N_{S}}\frac{z_{t}{\text{w}}^{T}{\text{y}}_{t}}{\left|{\text{w}}^{T}{\text{y}}_{t}\right|}\right|,$$ (7)

where| · |denotes the amplitude of a complex signal and${\text{y}}_{t}$isk−dimensional vector at the$t^{th}\;-$sample point.

Although the function defined in (7) is differentiable, convexity with respect towcannot be guaranteed. Thus, the best solution is selected out of a pool of different solutions found by several random initializations ofw. Typically, between 10 and 15 independent runs of the algorithm are done, and the solution with the largest MVL is selected as the best one. The complete set of spatial filters that defines the PCO demixing matrix${\text{W}}_{PCO}\in {\mathbb{R}}^{k\times k}$is found by solving (7) iteratively, out projecting the previously found filters from the solution space (Dähne et al., 2014;Waterstraat et al., 2017). It is important to mention that the data are firstly whitened by a matrixM. The column vectors of${\text{W}}_{PCO}$are sorted in decreasing order according to the MVL value found during the optimization procedure. Thus, the firstmcomponents from the resulting data projection in the PCO space${\text{X}}_{PCO}={\text{W}}_{PCO}^{\text{T}}{\text{X}}_{SSD}$correspond to the speech artifact sources.

#### 2.1.4 Signal reconstruction

The objective of the PCD pipeline is to denoise signals contaminated with the speech artifact. The artifact source estimation is just a proxy to facilitate data cleaning. Given that two spatial-filtering transformations are applied as a chained process, the PCD unmixing matrix${\text{W}}_{PCD}$which projects the raw data from the amplifier space to the artifact source space should be computed by taking into account the learned linear transformations${\text{W}}_{PCO}$and${\text{W}}_{SSD}$as follows:

$${\text{W}}_{PCD}=\text{M}\;{\text{W}}_{SSD}\left[\begin{array}{cc} {\text{W}}_{PCO} & 0\\ 0 & {\text{I}}_{\left(N_{c}-k\times N_{c}-k\right)} \end{array}\right],$$ (8)

whereIdenotes the identity matrix of dimension$N_{c}-k\times$$N_{c}-k$, andMis the$N_{c}\times N_{c}$whitening matrix applied to find the set of solution vectors to (7).

Once the unmixing matrix is computed, the mixing matrix that defines the forward model explained in (1) can be calculated based on its pseudoinverse:

$${\text{A}}_{PCD}={\text{W}}_{PCD}^{†},$$ (9)

where†denotes the Moore–Penrose pseudoinverse. Then the set of equations that define the linear forward and backward model is given by

$$\{\begin{array}{c} \text{X}={\text{A}}_{PCD}\text{S}\\ \hat{\text{S}}={\text{W}}_{PCD}^{T}\text{X} \end{array}.$$ (10)

Now, by low-rank factorization, the denoised signal can be reconstructed by zeroing from the mixing and unmixing matrices the firstmcomponents which were previously identified as artifactual. Thus, signal reconstruction is simply done by

$${\text{X}}_{denoised}={\tilde{\text{A}}}_{PCD}{\tilde{\text{W}}}_{PCD}^{T}\text{X},$$ (11)

where${\tilde{\text{A}}}_{PCD}$and${\tilde{\text{W}}}_{PCD}$represent the mixing and unmixing matrices of rank$N_{c}-m$, respectively.

Figure 2depicts how contaminated recordings are denoised by applying PCD, in the temporal, spectral, and phase domains. For illustration purposes, we artificially contaminated iEEG recordings with an audio signal (Recording space,Fig. 2a), resulting in iEEG signals with a strong peak in spectral power around F0 (Fig. 2acenter), together with a consistent phase relationship with the acoustic signal (Fig. 2abottom). After SSD is applied, only a few components have enhanced power spectra around F0 (Fig. 2bcenter), serving as a good starting point for the next step. Applying PCO in the SSD space gives us components maximally phase coupled with the recorded audio (Fig. 2cbottom). The components identified as artifactual will be discarded for signal reconstruction, thus achieving a denoised signal (Fig. 2d).

### 2.2 Simulated neural data

Given that it has also been observed acoustic-induced vibration artifact during stimulus presentation (Roussel et al., 2020), physiological gamma modulation simulations allow direct comparison of denoised data to well-defined ground-truth simulated neural signals, ensuring gamma activity solely linked to speech production. In this line, to benchmark our method’s denoising performance, we applied PCD on simulated neural data with added simulated speech artifact. This approach allows direct comparison of denoised data with the ground-truth simulated neural signals. Several simulations were conducted as explained hereafter. Briefly, we simulated recurrent networks of leaky integrate-and-fire (LIF) neurons (N = 5000, 80% excitatory and 20% inhibitory) to generate 100 physiological broadband γ-source activities, defined as the summation of absolute values of the excitatory and inhibitory currents entering the excitatory neurons population (Supplementary Fig. 1). The model captures key features of physiological LFPs, including (i) the entrainment of the low-frequency fluctuations to the external input, (ii) the internal resonance of broadband γ-oscillations driven by the mean rate of the external input (Supplementary Fig. 2), and (iii) phase–amplitude coupling between low- and high-frequency oscillations (Supplementary Fig. 3). We defined a single simulated speech artifact source$S_{a}\left(t\right)$assuming that it is identical to the produced audio signal, denoted asz in the PCD pipeline$\left(S_{a}\left(t\right)\approx z\left(t\right)\right)$. After adjusting what we called the artifact-to-physiological gamma ratio (AGR), we linearly projected the sources to the simulated recordings by the application of a random mixing matrix. Note that AGR can be thought as the inverse of the SNR. We tested the PCD pipeline in different simulation scenarios based on the expression of the external input$\nu_{signal}\left(t\right)\;$driving LIF neurons and the audio source$S_{a}\left(t\right)$(Supplementary Fig. 1). Details about the model and simulation settings can be found in theSupplementary Table S1.

#### 2.2.1 Simulation scenarios

Toy scenarios were created to stress the method under different predefined source conditions, as depicted bySupplementary Figure 1.

##### 2.2.1.1 Toy examples

We used toy examples for initial assessment of the effect of the PCD pipeline to remove the acoustic-induced artifact in*in-silico*neural signals (Supplementary Figs. 4–8). We used three scenarios for these toy examples, where$\nu_{signal}\left(t\right)$was defined as the superposition of the sustained and periodically modulated signal, as follows:

$$\nu_{signal}\left(t\right)=A_{s}+A_{p}\sin\left(2\pi f_{p}t+\varphi_{p}\right),$$ (12)

where$A_{s}$is the amplitude of the sustained input, and$A_{p}$,$f_{p},$and$\varphi_{p}$are the amplitude, the frequency, and the phase of the periodic signal, respectively.

In Scenario 1 (sinusoidal audio scenario, SAS), we synthetized the contaminating audio source$S_{a}\;$as a sinusoidal function with noise, as follows:

$$S_{a}\left(t\right)=A_{0}sin\left(2\pi F_{0}t+\varphi_{0}\right)+\eta \left(t\right),$$ (13)

where$A_{0}$is the amplitude,$F_{0}$is the fundamental frequency,$\varphi_{0}$is the phase, andη(t)is the white noise term.

In Scenario 2 (colored noise audio scenario, CAS), the audio source was synthesized by coloring the spectrum of the white noiseη(t), as follows:

$$S_{a}\left(t\right)=BP\left(t\right)*\sigma \eta \left(t\right)$$ (14)

where BP(t) is the impulse response of the 25^th^order Butterworth filter,σis the standard deviation, and*is the convolution operator.

In Scenario 3 (modulated colored noise scenario, MCAS), we mimicked the temporal profile of the audio in the Syllable Repetition Task (see*Triplet repetition task data*), applying the activation patternM(t)to the audio source in Scenario 2, which reads:

$$M\left(t\right)=\{\begin{array}{c} 1,\;\;t_{i}<\;t<t_{i}+\;0.5\\ 0,\;\;elsewhere \end{array};i=1,2,3$$ (15)

where$t_{i}$is the onset ofi−thsyllable, which lasts 0.5 s. Parameters used to define the audio signal in the different toys examples can be found inSupplementary Table S2.

##### 2.2.1.2 Realistic scenario

In this scenario (recorded audio scenario, RAS), we simulated 60 trials of speech-locked transient γ-source activity, feeding Gaussian signals in the network, as follows:

νsignal(t)=Ae−(t−μ)2(2FWHM2.355)2, (16)

where A andγare the intensity and temporal location modulation, andFWHMis the fullwidth at half maximum which controls the temporal focality of the modulation. Parameters were set to reproduce physiological speech-locked γ-modulation patterns (seeSupplementary Fig. 9) described in the literature (Chrabaszcz et al., 2019;Fischer et al., 2020;Moses et al., 2021). As audio source, we selected recorded audios of Parkinson’s disease patients during the Syllable Repetition Task. Three different audio signals were selected accordingly to the spectrogram profiles, for example, nonstationary of the pitch (Supplementary Fig. 10). Across trials, the same audio signal was mixed by changing the linear mixing matrixAinEq. (1).

### 2.3 Neural real data

In total, 54 English-speaking patients undergoing an awake DBS implantation surgery consented to participate in an intraoperative speech task at the University of Pittsburgh (IRB Protocol #PRO13110420). Patients were instructed to repeat consonant–vowel syllable triplets that were played on their earphones. Specifically, the basis set of phonemes included four consonants (/v/, /t/, /s/, /g/) and three cardinal vowels (/i/, /a, /u/) with distinctive tongue positions and acoustic properties. Then, 1 to 4 recording sessions of up to 120 syllable triplets each were performed by participants. Sessions differed regarding the neural recording modalities registered. Electrocorticography (ECoG) was always registered through 1 or 2 strips of 53 or 64 contacts targeted to cover the left ventral sensorimotor cortex, superior temporal gyrus, and inferior frontal regions. Microelectrode recordings (MER) and LFPs from a macro ring located 3 mm above the microelectrode tip were registered during subcortical target mapping, for participants undergoing subthalamic nucleus (STN) or Globus Pallidus Internus (GPi) DBS. LFPs registered from the DBS lead were recorded from the STN, GPi, and ventral intermediate nucleus (VIM). The produced audio signal was recorded by a directional microphone placed near the patient’s mouth. Data were time-aligned, preprocessed, low-pass filtered at 500 Hz, down-sampled to 1 kHz, and saved as a FieldTrip (Oostenveld et al., 2011) object for subsequent analyses. For more information about the dataset, we refer the reader toBush et al. (2022).

### 2.4 PCD algorithm implementation

F0 may vary between trials of the same participant. As a result, the phase relationships between the artifact source and the neural recordings change across trials (Supplementary Fig. 12). To better track F0 changes, the PCD pipeline was applied on a trial-wise basis. Data were high-pass filtered above 2 Hz and notch filtered at 60 Hz and its three first harmonics. Given that the artifact was observed only during overt speech production times, epochs around the produced speech onset were extracted for fitting the model. Under the hypothesis that the artifact is introduced in the acquisition chain, the different synchronized brain recording modalities (MER-LFP + ECoG or DBS-LFP + ECoG) were combined to form a unique data matrix. That is, the signals from all available brain recording modalities were treated as a unique sensor space.

For every trial, the model was fitted as follows. First, the audio signal was used to estimate the SAFB, and thus the noise and signal band were accordingly defined (Box 1- step 1). Data preparation proceeded, which included applying the Hilbert transform to find the analytic representation of the signal, as well as data whitening. The audio signal was z-scored for normalization purposes and used as the best guess of the artifact source. The SSD algorithm was applied to the real part of the signal. The final number of SSD components to keep (*k*) was automatically selected based on the participation ratio (PR), defined as follows:

$$PR\;=\;\left\lfloor \frac{{\left(\Sigma_{i}\lambda_{i}\right)}^{2}}{\Sigma_{i}\lambda_{i}^{\;\;2}}\right\rfloor ,$$ (17)

where$\lambda_{1}\;\geq \lambda_{2}\geq \ldots \geq \lambda_{N_{C}}$are the eigenvalues resulting from solving SSD which account for the SNR participation of each SSD component. The PR has been shown to be a reasonable measure of neural dimensionality in PCA (Gao et al., 2017). PCO was applied on data projected onto the SSD space, extracting one component at the time. Every component resulted in a mean vector length (MVL) value, such that${\text{MVL}}_{1}\geq {\text{MVL}}_{2}\geq \ldots \geq {\text{MVL}}_{k}$. Once both spatial-filtering methods were learned, the unified unmixing and mixing matrices that described the PCD pipeline were computed (Box 1– step 4).

The identification of the artifactual sources was also automatically done by finding the elbow in the trace of the MVL value across components, that is the point at maximum curvature differentiating the contribution of strong and weak components. Those components showing the highest MVL values were identified as artifactual.

Considering that further analysis would be done on the data, a wider epoch starting 6 seconds before and ending 6 seconds after the produced onset was used when applying the learned matrix transformation.

### 2.5 Traditional methods for denoising

The performance of PCD was compared against the traditional denoising method based on spatial filtering. In the following, each of the baseline methods is presented.

#### 2.5.1 CAR as a spatial-filtering algorithm

Common average reference (CAR) is a spatial-filtering method that subtracts the common noise at every electrode, calculated as the average of all recordings. The data re-referenced via CAR are calculated as follows:

$$\begin{array}{l} X_{CAR}=X-\bar{X}\\ \;\;\;\;\;\;\;\;\;\;\;\;=X-\frac{1}{N}JX\\ \;\;\;\;\;\;\;\;\;\;\;\;=[I-\frac{1}{N_{c}}J]X\\ \;\;\;\;\;\;\;\;\;\;\;\;=W_{CAR}X, \end{array}$$ (18)

where$N_{c}$accounts for the number of electrodes and$\text{J}={\left[{\text{1}}^{N_{c}}\right]}^{T}$is an$N_{c}\times N_{c}$matrix of ones.

With this formulation, it is easy to see that CAR can be thought of as a spatial-filtering method which implies a linear transformation of the data.

Given that CAR takes the average across channels, data structure is not critical in this matter. The matrix transformation was applied in the continuous data for each type of channel recording.

#### 2.5.2 The PCA + ICA pipeline

Independent component analysis (ICA) assumes that the sources linearly mixed in (1) are independent. Such an assumption is true for many artifacts that appear in brain recordings, such as electrocardiography or electromyography. In order to ensure that independent sources are extracted from the data, non-Gaussianity of${\text{W}}^{T}\text{X}$is maximized. Here in particular, ICA is implemented using Picard (Ablin et al., 2018), a fast algorithm to solve the maximum likelihood estimation ICA formulation. This ICA implementation was chosen since it is known to result in robust components estimations in cases where the sources are not completely independent.

Basic data preprocessing was done before applying ICA. A temporal filtering above 2 Hz was implemented in order to remove low-frequency drifts, which can negatively affect the quality of the ICA fit (Winkler et al., 2015). Then data were z-scored and PCA was applied in order to both feed ICA with whitened data and to reduce the dimensionality of the problem. The number of PCA components was automatically selected by setting the explained variance to 0.99. As in the PCD pipeline, model fitting was done using the windowed data within the produced audio epoch, combining, if exists, the different brain recording modalities. For the sake of comparison with PCD, artifactual sources identification was done based on the circular mean of the phase difference. Those components showing the highest phase-locking values were selected as artifactual. Denoising via low-rank factorization was applied in the wider epochs, as done for PCD.

### 2.6 Metric for performance assessment

#### 2.6.1 Performance assessment in toy examples

Given that in toy examples we simulated only one artifactual source, we expected the PCO to find only one component with high coherence with the audio (i.e. MVL) in the PCO space.

To assess the performance of the denoising pipeline, we calculated time-, frequency-, and phase-domain metrics that estimate the agreement between the ground-truth${\text{X}}_{gt}$and cleaned dataX, as well as between the truez(t)and estimated artifactual source$\tilde{z}\left(t\right)$.

For each channel, we compared the similarity in the time domain of the neural data$\left(\text{X}\;vs.{\text{X}}_{gt}\right)$as follows:

$$\chi^{2}=\;\frac{1}{T}\sum_{t\;=\;1}^{T}\frac{{\left(\text{X}-{\text{X}}_{gt}\right)}^{2}}{\sigma^{2}\left({\text{X}}_{gt}\right)},$$ (19)

whereTis the duration of the simulation and$\sigma^{2}\left(\cdot \right)$is the variance.χvalues have been converted to a logarithmic scale for visualization purposes (Supplementary Figs. 5,7).

To assess the fidelity of the estimated speech-induced artifact$\left(\text{z}\;vs.\;\tilde{\text{z}}\right)$, we used the magnitude coherence estimate (MSCE) and the consistency of the phase difference based on the PLV. The MSCE returns values between 0 and 1 indicating how similar two signals are at each frequency, as follows:

$$\text{MSCE}\left(f\right)=\frac{{\left|P_{\text{z},\tilde{\text{z}}}\left(f\right)\right|}^{2}}{P_{\text{z},\text{z}}\left(f\right)P_{\tilde{\text{z}},\tilde{\text{z}}}\left(f\right)},$$ (20)

wherePstands for the power spectral density. The PLV is a metric between 0 and 1 that quantifies the phase agreement between two signals, as follows:

$$PLV\;=\left|\sum_{t\;=\;1}^{T}\frac{e^{i\left(\phi_{\text{z}}\left(t\right)-\phi_{\tilde{\text{z}}}\left(t\right)\right)}}{T}\right|,$$ (21)

whereϕdenotes for the instantaneous phase.

In addition, in the RAS examples, we assessed the similarity of the true and estimated audio source by computing the one-dimensional statistical parametric mapping (SPM1d,https://spm1d.org/). By mean of SPM1d, statistical differences at the sample level were assessed. Finally, to evaluate the goodness in the source recovery of the method at a global level, the Mean Square Error (MSE) was also computed. To account for a bounded MSE value, each signal was first divided by its maximum value before computing the error. As such, the closer to zero the normalized MSE is, the most similar the two signals are.

#### 2.6.2 Neural preservation assessment

Principal Component Analysis (PCA) is a dimensionality reduction technique that identifies an ordered set of orthogonal directions that captures the greatest variance in the data. It is widely accepted and used in the neuroscience community for analyzing neural population activity (Cunningham & Yu, 2014). The low-dimensional space identified by PCA captures variance of all types, including noise. Such data representation can be thought of as the Cartesian coordinate basis describing subspaces in which the data lie (Chung & Abbott, 2021). Thus, for assessing neural preservation after applying denoising methods, the PCA low-dimensional space was utilized. Subspaces describing the same geometry as the ground-truth data should be found after denoising if neural preservation is achieved. Thus, for every denoising pipeline, as well as the ground truth and the noisy data, PCA was fitted independently. The learned loading and scores were plotted for each decomposition made on the first three principal components (PC).

To quantify the preservation of the physiological signal, we measured the degree of similarity between the PCA loadings ($\text{L}\in {\mathbb{R}}^{N_{c}\times N_{c}}$) of the ground-truth data and the resulting data after each denoising pipeline was applied, using the cosine similarity (CS) (Koay et al., 2022). CS measures the extent to which two vectors point in the same direction. In this way, CS was used as a tool to quantify the degree of neural preservation after applying a denoising pipeline. Given that PCA loading signs are arbitrary, bounded CS between 0 and 1 can be found by taking the absolute value of the cosine similarity, as follows:

$$CS_{i}\;=\;\;\left|\frac{{\text{L}}_{i}^{gt}{\text{L}}_{i}}{\left\|{\text{L}}_{i}^{gt}\right\|\;\;\left\|{\text{L}}_{i}\right\|}\right|,$$ (22)

whereidenotes the index of a given loading vector,${\text{L}}_{gt}$indicates the PCA loading matrix of the ground-truth data${\text{X}}_{gt}$,Lstands for the PCA loading matrix of a given denoised data${\text{X}}_{denoised}$, and|| · ||and| · |denote the$\ell_{2}-$norm and the absolute value operators, respectively. The closer CS is to 1, the more similar the two vectors are. Thus, a good denoising pipeline from the neural data preservation point of view should be the one from which PCA loading vectors resemble the same directions as the data with only true gamma modulation.

#### 2.6.3 Intertrial coherence for speech artifact quantification

The speech artifact level at each electrode was computed based on the intertrial phase consistency (ITPC) (Cohen, 2014), following the same framework proposed and used inBush et al. (2022). That is, the audio and the neural signals were band-pass filtered between 70 and 240 Hz, that is, within the plausible SAFB range. Then considering the complex representation of the neural data for a given channely=x+iℋ(x)and the audio signalzat a given triale, the phase between these two quantifies can be measured by

$$\varphi_{e}=\;\frac{1}{\left||\;\text{​}\text{y}\;\text{​}||\;||\text{​}\;\text{z}\;\text{​}|\right|}\sum_{t}y_{\text{t}}z_{t}.$$ (23)

At the end of this procedure, all the phases across the$N_{t}$trials are arranged on a vector$\varphi =\;[\varphi_{1},\ldots ,\varphi_{{\text{N}}_{\text{t}}}]$. If there is intertrial phase consistency, the mean value of**ϕ**across trials (〈**ϕ**〉) will be different from 0, and thus it can be quantified as follows:

$$ITPC\;=\frac{\;\left|\langle \varphi \rangle \right|}{std\left(\varphi \right)},$$ (24)

wherestd(·)stands for the standard deviation. It has been found that ITPC values equal or above 3.08 indicate that the presence of the speech artifact on that given electrode is significant, and thus the electrode must be considered contaminated (Bush et al., 2022). Here ITPC was used to assess the level of audio contamination an electrode has either before or after a denoising pipelines was applied.

#### 2.6.3 Artifact presence quantification: definition of homogeneity, strength, and clean electrode gain

To understand denoising performance with respect to the characteristic of the artifact presence across electrodes, we defined indices to quantifying the artifact homogeneity and strength across electrodes, as follows.

Artifact homogeneity should quantify the consistency of the artifact presence across electrodes. Let us denote$\vartheta =\left[ITPC_{1},\ldots ,ITPC_{N_{C}}\right]$as a vector with the stored ITPC value found per each electrode. Considering thatn-dimenstional unit vectors have variance between 0 and1n, following the idea proposed byUmakantha et al. (2021), we define the artifact homogeneity as follows:

$$artifact\;homogeneity=1-var\left(\frac{\vartheta }{\left\|\vartheta \right\|}\right)N_{c},$$ (25)

that is, a value between 0 and 1, for homogeneous artifact presence and non-homogeneuous artifact presence, respectively.

To summarize the presence of the artifact, we defined the artifact strength by taking the mean ITPC value found across electrodes:

artifact strength=mean(ϑ). (26)

In addition, we computed the clean electrode gain by taking the relative change of the clean electrode percentage before ($\%CE_{denoised}$) and after ($\%CE_{raw}$) applying a denoising method, that is

$$gain=\%CE_{denoised}-\%CE_{raw}.$$ (27)

### 2.7 Deep learning model for consonant identification

In the case of BCI for speech prothesis, the denoised data will most probably be used to feed a brain decoding algorithm, reason from which it is important to evaluate the impact of each denoising method as a preprocessing step in a speech decoding pipeline. Here, a densely connected convolutional neural network (DenseNet) (Huang et al., 2017) was trained to classify the consonants from neural signals (ECoG). For each syllable, original/denoised ECoG signals were spectrally filtered into 7 frequency bands ranging between 0 to 250 Hz. These syllable-level neural data were used as training set. We then extracted the perceptual linear prediction (PLP) (Hermansky, 1990) features from the corresponding audio recordings. Both PLP features and consonant identities were used as training labels. Our DenseNet model was designed to first map neural signals into PLP spectra and then predict the consonant class from the PLP space. For that purpose, the mean-squared-error loss of PLP feature prediction and the cross-entropy loss of consonant classification were jointly optimized during model training. We used 5-fold cross-validation while measuring model performance, withholding a different 20% partition from the training data as test set each time. Separate models were trained for each subject and each data type, resulting in 12 different models (3 subjects, 4 data types: Raw, CAR, ICA, PCD). During testing, reserved ECoG data were fed into the trained model, and the accuracy was measured based on the consonant predictions.

## 3 Experiments and Results

### 3.1 Method benchmarking on in-silico data

#### 3.1.1 Algorithm stress test on simulated data: parametric sweep and performance evaluation

We applied the PCD pipeline on simulated data using the speech artifact$S_{a}\left(t\right)$as audio signal$\left(S_{a}\left(t\right)\approx z\left(t\right)\right)$. Given that we simulated only one artifactual source, we expected the PCO to find only one component with high coherence with the audio (i.e. MVL) in the PCO space. The quality of denoised data was compared against the ground truth by means ofEq. (19), while the similarity of the retrieved artifactual source to the original one was computed usingEqs. (20)and(21).

To stress the PCD pipeline in the different scenarios, we evaluated performance during a sweep of key parameters in the toy SAS and CAS examples. While for the SAS scenario AGR [-100, 30] dB,F0[70, 180] Hz and$N_{c}$[3, 100] were varied, in the CAS scenario we sweptΔF [2, 16] Hz, the filter order[3, 27] and the simulation duration [0.5, 3.5] s.

Results showed that for pure sinusoidal artifacts, PCD perfectly removed the artifact regardless of the AGR, fundamental frequency, and number of channels (Supplementary Figs. 4, 5). For the colored noise artifact, simulations suggested a small decrease of performance with broadband artifacts, which is consistent with known limitations of SSD (Supplementary Figs. 6, 7). Finally, PCD yielded robust performances when tested with modulated colored noise artifact simulations (Supplementary Fig. 8).

Similarly, in the case of RAS scenario, PCD reliably recovered the artifactual source, as can be observed in the time, frequency, and time–frequency profiles of both the true (Fig. 3a) and estimated (Fig. 3b) artifact sources. Across trials, no differences were found at any sample point between the true and the estimated artifact sources, as assessed by SPM1d (Fig. 3c). The median-normalized MSE yielded value across trials was$1.5\;x\;10^{-3}$. The estimated artifactual source had high coherence with the true artifactual source at frequencies around F0 (mean MSCE > 0.97,Fig. 3d), independently of differences in the AGR (Supplementary Fig. 11). Finally, the estimated source was either in phase or antiphase relationship with respect to the true source (Fig. 3e). Similar results were found for the other realistic simulations (Supplementary Fig. 11), showing that independently of the pitch modulation (F0 nonstationarity) and the AGR value, the artifactual source could be retrieved. Nevertheless, as may be expected, stronger artifacts (high AGR) were more accurately extracted by the method, rendering higher PLV of the extracted component with the simulated artifact source (Supplementary Fig. 11)**.**

### 3.2 PCD removes the speech artifact while preserving simulated γ activity

Next, we compared the performance of PCD with CAR and ICA using simulated data under the realistic scenario (RAS). For a fair comparison, all three methods were implemented on a trial-wise basis and components identified by ICA were scored according to their phase-coupling value against the recorded audio. We evaluated performance in terms of the capacity of each method to retrieve the temporal, frequency, and phase information of the simulated ground-truth neural data (Fig. 4a–c).Figure 4shows the simulated neural activity without the artifact source (Artifact free), when linearly combined with the artifactual source (Contaminated), and the resulting denoised signals from each method (CAR, ICA, PCD). PCD outperformed the other methods in terms of its ability to clean the data and retrieve the simulated brain signals, as can be observed in the time (Fig. 4a), frequency (Fig. 4b), and phase (Fig. 4c) domains. In this simulation, CAR produced traces very similar to the contaminated signal, while ICA attenuated the artifact to some extent. Interestingly, PCD completely removed the narrow-band component induced by the speech artifact while preserving gamma modulation observed in the simulated brain signals.

To assess the preservation of physiological brain signals after denoising, we compared the neural embedding defined in the subspace spanned by the first three PCA components (PC subspace). Note how the PC embedding is distorted in the contaminated signals, as compared with the artifact-free signals (Fig. 4d). CAR resulted in an embedding indistinguishable from the contaminated signals (Fig. 4d), consistent with the time, frequency, and phase analyses. Interestingly, while ICA was able to attenuate the speech artifact, the PC embedding was different from that for the artifact-free signals, indicating that ICA distorted the underlying physiological sources. Here, PCD was the only algorithm that completely removed the artifactual source, as assessed by the signals’ power spectrum (Fig. 4b) and phase-coupling (Fig. 4c) plots, while simultaneously preserving the underlying physiological activity, as evidenced by the indistinguishable PC embedding to that of the artifact-free signals (Fig. 4d). To quantitively assess the similarities to the ground-truth PC embedding, and thus provide a proxy for the preservation of the underlying neural signal after denoising, we used the cosine similarity metric of the three first components (Eq. (22)). The mean resulting value was of 0.79, 0.74, and 0.99 for CAR, ICA, and PCD, respectively, while for the contaminated data this value was 0.79. Lower CS value for ICA than the contaminated data could be explained by the fact that ICA is changing the embedding even more drastically than the artifact itself.

### 3.3 Denoising PCD performance in acoustic contaminated iEEG data

We applied the PCD pipeline to intracranial recordings of 54 patients undergoing DBS implantation surgery who participated in an intraoperative speech task (seeSection 2.3). As we previously described, around 40% of channels in this dataset show speech-induced vibration artifact (Bush et al., 2022).

Considering that the source of the speech artifact is the same across different types of electrodes, the denoising pipeline was applied to all available iEEG recordings together (LFP from ECoG, the DBS lead, and the ring and tip contact of the microelectrode). Given that phase relationships between the recorded audio and the neural recordings are not consistent across trials (Supplementary Fig. 12), data cleaning occurred in a trial-wise manner. The resulting denoised signals were compared with those obtained after applying CAR and ICA as inFigure 4. The number of components to be removed was automatically selected based on the elbow detection on the MVL values.

To assess denoising performance, we used the distribution of ITPC with the audio (Eq. (24)), (Fig. 5a) and the percentage of electrodes without significant ITPC (Fig. 5b) before vs. after applying a denoising framework (Raw vs. CAR, ICA, PCD). Results show that CAR exacerbated the artifact, shifting the ITPC distribution toward higher values (Fig. 5a), whereas ICA and PCD reduced ITPC values and increased the percentage of electrodes without significant coherence with the audio (Fig. 5a–b). However, as shown next, the low coherence values for ICA were due to an aggressive removal of all high-frequency components (artifactual and physiological).

To illustrate the method’s performances, time–frequency plots are shown from two electrodes of the same participant and trial. Electrode ecog_1_49 has a strong artifactual narrow-band component after speech onset (Fig. 5ctop panel), while ecog_1_59 has a characteristic physiological gamma modulation around the time of speech onset (Fig. 5cbottom panel). Note that the speech artifact is either exacerbated or artificially introduced after applying CAR due to the presence of the artifact on other electrodes (Fig. 5c– CAR). Interestingly, ICA abolished the physiological gamma modulation observed in ecog_1_59, whereas PCD preserved this activity while simultaneously removing the narrow-band component (Fig. 5c). A similar plot, but for electrodes with strong artifact component also in the first harmonic of F0, is presented inSupplementary Figure 13, showing that the method can reduce the presence of the artifact also in harmonics F0 frequencies.

It is of particular interest to analyze the high-gamma profiles before and after applying each denoising method. To this end, we extracted high-gamma power time locked to the auditory stimulus onset and the speech onset, for electrodes located in auditory cortex (superior temporal gyrus,Fig. 5d) and motor cortex (precentral gyrus,Fig. 5e), respectively. The gamma response shown in the auditory cortex during auditory stimulus presentation was unaffected by the artifact; however, it was greatly attenuated by ICA (Fig. 5d). Similarly, ICA also abolished the gamma response during speech production for the electrodes shown over motor cortex (Fig. 5e).

To further explore the differences in performance across methods, we studied the artifactual sources retrieved by CAR, ICA, and PCD. For the case of ICA and PCD, the first component (which maximum phase-locking value with the audio) is shown. In addition, the number of removed components by these two methods was analyzed. Although in most of the cases between 1 and 4 components were removed by both ICA and PCD (Supplementary Fig. 14), the artifactual sources retrieved by ICA do not resemble a speech-induced artifact (Fig. 6). As expected, CAR component, calculated by taking the mean across channels, also does not resemble the vibration artifact.

Additionally, to explore whether the variability in denoising performance can be explained by characteristics of the artifact across electrodes, we evaluated the relationship between the relative gain of electrodes by each denoising method (Eq. (27)), artifact strength (Eq. (26)), and artifact homogeneity (Eq. (25)). Results are shown inFigure 7a. Note that recordings with negative gain (i.e. increased in percentage affected electrodes) are shown in red. CAR has good denoising performance only for highly homogeneous artifacts with a mild-to-moderate artifact strength (Pearson correlation${\text{r}}_{\text{gain}-\text{homogeneity}}=0.59$, p < 0.0001;${\text{r}}_{\text{gain}-\text{ strength}}=0.23$, p = 0.006) and can outperform the other methods under these conditions (e.g. Subject A, blue line circles inFig. 7a). Note that CAR can introduce artifact to clean data (e.g., Subject C, black line circles inFig. 7a). For ICA, stronger artifacts with mild-to-moderate homogeneity resulted in higher gain of clean electrodes, as assessed by ITPC (${\text{r}}_{\text{gain}-\text{strength}}=0.57$, p < 0.0001;${\text{r}}_{\text{gain}-\text{homogeneity}}=0.24$, p = 0.003). Interestingly, PCD gain also increases with higher artifact strength but is not significantly correlated with artifact homogeneity (${\text{r}}_{\text{gain}-\text{strength}}=0.50$, p < 0.0001;${\text{r}}_{\text{gain}-\text{homogeneity}}=-0.12$, p = 0.14), as illustrated by Subject B (gray line circles inFig. 7a).

Finally, we assessed the effect of applying each denoising method as a preprocessing step on decoding performance of a densely connected convolutional neural network (DenseNet) (Huang et al., 2017) for consonant decoding. We tested the decoding method in three different cases: (i) CAR and ICA gain is greater than PCD gain (Subject A), (ii) PCD outperforms CAR and ICA (Subject B), and (iii) data have no artifact (Subject C).Figure 7bshows that consonant classification accuracy is similar or better when PCD is applied as compared with classification on raw data. Conversely, ICA always decreases classification accuracy, despite increasing the number of electrodes bellow the significant ITPC threshold. Interestingly, for Subject A although the number of contaminated electrodes increased after CAR, the consonant classification accuracy improved, suggesting that decoding capacity in this subject might be partially driven by the artifact (Fig. 7b-Subject B).

## 4 Discussion

In the last decade, the use of iEEG for clinical and research purposes has increased all over the world (Mercier et al., 2022). The new developments and technologies with respect to electrode designs have opened the door for studying brain activity with higher spatial and temporal resolution. The study of human processing and speech synthesis has gained tremendous advantages by data recorded within the brain, as shown by the latest findings in speech decoding (Moses et al., 2021). Nevertheless, it has been recently discovered that acoustic-induced vibrations can affect the quality of iEEG signals recorded speech perception and/or production tasks. This recently described acoustic-induced artifact (Bush et al., 2022;Roussel et al., 2020) overlaps in time and frequency with gamma-band activity and can be assessed by the phase relationship with the played or spoken audio (Bush et al., 2022).

Given that the presence of such artifact can compromise the reliability of speech-related analyses, including those used in BCI development, in the present work we have presented a data-driven spatial-filtering approach for safety removal of speech-induced vibration artifacts in the iEEG.

To raise awareness to the community, we have evaluated the performance of two traditional and commonly used spatial-filtering methods for artifact removal. Through our experiments, we demonstrated that traditional spatial-filtering approaches are not appropriate for denoising the speech artifact (Figs. 4,5). Specifically, we showed that CAR exacerbates the presence of the speech artifact when it is heterogeneous across recording channels, "subtracting in" the artifact to otherwise unaffected channels (Fig. 5- CAR). As expected, CAR only showed good performance when the vibration artifact had a homogeneous representation across electrodes (Fig. 7a- CAR). Nevertheless, the vibration artifact is a channel-specific type of noise, and as such, while CAR can be part of traditional preprocessing pipelines, it should not be used without proper data analysis and evaluation of the artifact. For the case of ICA, it can be observed that while the method reduces the coherence of neural signals with the audio (Fig. 5a- ICA), this comes at the cost of a strong degradation of physiologicalγ-band modulations (Fig. 5d, e- ICA), which ultimately results in a reduction of speech-decoding performance from neural data (Fig. 7b- ICA). Interestingly, when analyzing simulated data, the cosine similarity of PCA embeddings to the ground-truth data was lower after applying ICA than for the contaminated data. This demonstrates that ICA can degrade the underlying neural data more aggressively than the artifact itself. Although artifactual ICA components were selected based on the phase relationship with the audio, this strong data degradation could indicate that ICA fails in identifying and differentiating true gamma modulation from the speech artifact. The frequency band overlap between these two signals compromises the strong independence assumption of ICA, foreseen for a reliable method performance.

In recent years, data-driven spatial-filtering methods have been introduced as re-referencing schemes (Cohen, 2017;de Cheveigné, 2020;Schaworonkow & Voytek, 2021). Such is the case of SSD, an effective method to increase the SNR for narrow-band components, in which not only the central frequency of the band of interest is enhanced but also its harmonics (Schaworonkow & Voytek, 2021): a characteristic that is particularly suitable for denoising speech-induced artifacts. Given that the speech contaminations can be assessed by means of coherence with audio channels (ITPC) (Bush et al., 2022), PCO (another data-driven spatial-filtering method) is ideally suited to decompose the acoustic artifact source from brain recordings. By combining these methods, we developed PCD, a novel data-driven algorithm for denoising brain recordings contaminated with acoustic-induced artifacts (Figs. 1,2). Here, we show that PCD can retrieve the speech artifact while preserving physiologicalγ-band modulations at overlapping frequencies (Figs. 3,4). Through extensive simulations, we show that PCD works for different number of channels (from 3 to 100), different ratios of artifact to neural sources amplitude (from -100 to 30 dB), and across different durations of simulated artifact (from 0.5 to 3.5 s), although it is sensitive to the SSD filter parameters such as the bandwidth around F0 (ΔF) and the filter order (Supplementary Figs. 5, 7). Experiments in realistic scenarios showed that PCD can retrieve the artifactual source regardless of the pitch modulation of the audio signal (Fig. 3andSupplementary Fig. 11), demonstrating robustness with respect to F0 changes. Numerical experiments at different level of contamination, simulated by changing the AGR value, showed that artifact estimation was always successful, with only a small drop in the phase-locking value at lower levels of the artifact for some audio types (Supplementary Fig. 11- audio types 2 and 3). The relationship between artifact strength and PCD denoising capability was also shown in real data experiments as assessed byFigure 7. These results show that even in cases in which the artifact presence might be low, the use of PCD can help in data cleaning. Nevertheless, the stronger the artifact is, the better the performance of the method will be (Fig. 7- PCD).

In addition, experiments in real data showed that PCD can diminish the number of artifactual electrodes without distorting the underlying neural response (Figs. 5,7). High-gamma profiles show that PCD (but not ICA) preserves physiological gamma responses (Fig. 5d, e), indicating PCD represents a more reliable denoising method for these speech artifacts than other traditional pipelines. This finding was also replicated when inspecting the detected artifactual source (Fig. 6) and when testing each denoising method as a preprocessing step of a deep learning network for consonant detection (Fig. 7b). Here, it is interesting to mention that in the three evaluated cases, regardless of the electrode gain performance, ICA always resulted in the lowest consonant decoding accuracy, suggesting that relevant neural activity that encodes speech has also been removed during the ICA denoising.

PCD was designed by considering the characteristics of the speech-induced vibration artifact. As such, the algorithm first applies SSD to enhance the AGR in a frequency band centered at F0 (SAFB). Given that we do so in a trial-wise manner, the changes of F0 across trials are tracked. In addition, for participants with low F0, at which it first harmonics of their F0 may also be in the high-gamma range, the use of SSD also ensures an enhancement of the spectral peak for the first harmonic of F0 (Schaworonkow & Voytek, 2021). Although not perfectly, the harmonics of F0 can be reduced by means of PCD (seeSupplementary Fig. 13). Retaining those components at which the AGR in the SAFB is maximized reduces the dimensionality of the problem enough to proceed with the identification of those components phase locked with the audio. Given that PCO is a nonconvex optimization problem, the lower the dimensionality of the search space is, the faster the method converges. As in the traditional PCA+ ICA pipeline, the use of SSD before PCO prepares the data space to maximize the chances of success in the artifact identification.

Nevertheless, the PCD method has several underlying assumptions. First, it assumes the speech artifact is a narrow-band component around the fundamental frequency of the participant’s voice, which must be estimated for each participant from audio recordings. Second, it assumes the artifact source is common across electrode modalities, thus allowing the combination of all recording modalities which maximizes the chance of extracting the artifact source (Cohen, 2017). While this might be counterintuitive, the artifact is likely due to mechanical vibrations of cables and connectors along the recording chain (Roussel et al., 2020), which can affect different recording modalities in the same way. Third, we assume that the recorded audio is a good estimation of the true artifactual source (i.e., there is no spectral distortion or delay between audio and artifact). While this is a strong assumption, currently there is no better proxy for the artifactual source than the recorded audio. Violations of this assumption may explain the difference between simulated (Figs. 3,4) and real data performance (Fig. 5).

The current implementation of PCD has several limitations. (i) Performance declines for broadband artifactual sources (Supplementary Fig. 7), a limitation inherited from SSD, which can enhance the power only in narrow frequency bands (Nikulin et al., 2011;Schaworonkow & Voytek, 2021). This limitation could also partially explain the differences in performance between simulated and real data. (ii) The method does not account for systematic distortions between the recorded audio and the speech artifact. Modeling such distortions might be a promising approach to further improve the method's performance in future studies. (iii) PCD is computationally expensive given that it involves solving a nonconvex optimization problem, thus requiring several runs until it converges to the best solution (Waterstraat et al., 2017). As such, PCD for online BCI applications would require further ad-hoc implementations to speed up the optimization process. (iv) The method must be applied in a trial-wise manner given that the artifact's phase relationship with the audio strongly varies across trials (Supplementary Fig. 12). For this reason, the method was fitted to individual speech production epochs to optimally estimate the artifact and then a wider window was applied to avoid introducing discontinuities that could affect subsequent analyses. Applying PCD per trial has the additional advantage of reducing memory requirements and computational cost.

Despite the orders of magnitude greater resolution of intracranial recordings compared with scalp EEG signals, the potential for induced speech artifacts is still a significant concern (Bush et al., 2022;Roussel et al., 2020). Critical to BCI development using intracranial recordings from overt speech production is the recognition that these artifacts can hamper training of robust machine learning models, which may be biased and fail when used for speech-impaired patients in real-life applications. Although the origins of the acoustic-induced artifact are still unknown, care must be taken when recording neural data to diminish the potential sources of the vibration artifacts. Evaluating different reference schemes may help in some cases (Mercier et al., 2022), but ultimately the presence of acoustic-induced artifacts should be always assessed. If contamination is found, signal preprocessing methods should verify not only that artifactual components are removed or attenuated, but also that neural signals are retained. The PCD method may facilitate the building of BCI decoding models upon reliable neural data by avoiding artifactually driven performance. Additionally, PCD was designed specifically to mitigate loss of signal quality we observed using standard preprocessing methodologies for speech artifact denoising. Using simulated data, we showed that when PCD's assumptions are satisfied, the method can completely remove the speech artifact while preserving speech-relatedγ-band modulations. For real data, for which the underlying assumptions might not be strictly satisfied, PCD still achieves significant reductions of the strength and extent of the speech artifact. The consonant decoder results obtained with PCD-denoised signals illustrate the practicability of this method for potential improvements in the reliability of BCI frameworks. Moreover, although this study focused on denoising speech artifacts in intracranial recordings collected intraoperatively, PCD can in principle be applied to remove any narrow-band artifact from electrophysiological data, including sEEG and EEG, if an estimate of the artifactual source is available. Further research is needed to assess the performance of PCD in different settings such as noninvasive EEG, and for different types of narrow-band artifacts. When the artifact is known to be spread across different electrodes or electrode modalities, we recommend pooling all channels to a common data matrix from which the artifactual components should be extracted.

## Computing Environment

Data simulation and numerical experiments were conducted in Matlab 2020b. We used custom-based scripts (BML toolbox;https://github.com/Brain-Modulation-Lab/bmlbmlbmlbmlbmlbased on the Fieldtrip library (https://www.fieldtriptoolbox.org). We used online available implementations of SSD (https://github.com/svendaehne/matlab_SSD) and PCO (https://github.com/neurophysics/PCO_matlab). For measuring the phase-locking values and phase differences, the CircStast toolbox (https://www.jstatsoft.org/article/view/v031i10) was utilized. In addition, Rstudio was used to compute the ITPC. For computing the simulations, we used the publicly available C-optimized implementation (http://senselab.med.yale.edu/ModelDB/ShowModel.asp?model=152539)^33^. We computed the MSCE and the consistency of the phase difference by using the built-in MATLAB*mscohere*function and*circ_r*function in the CircStat Toolbox^51^, respectively. We used the RainCloud library^52^to compare distributions of data (https://github.com/RainCloudPlots/RainCloudPlots#read-the-preprint).

## Supplementary Material

Supplementary Material

## Data Availability

The data that support the findings of this study are available upon reasonable request. A formal data sharing agreement is required. The code for the PSD algorithm is available online athttps://github.com/Brain-Modulation-Lab/PCD
