# Supplementary material for: A supervised data-driven spatial filter denoising method for speech artifacts in intracranial electrophysiological recordings
Source: Imaging Neurosci (Camb). 2024 Oct 1;2:imag-2-00301. doi: 10.1162/imag_a_00301 (PMC12290705; doi:10.1162/imag_a_00301)
Supplement: Supplementary Material [file imag_a_00301-supp.pdf]

Supplementary Text

Details about the simulation approach

1. The simulated neural source model

We simulated a recurrent network of leaky integrate-and-fire (LIF) neurons (N = 5000): 80% excitatory neurons I with AMPA-like synapses and 20% inhibitory neurons (I) with GABA-like synapses (Buzsáki & Wang, 2012; Meneghetti et al., 2021) (Supplementary Fig 1, Supplementary Table 1 for parameters). The connectivity architecture is sparse and random with an average connection probability between any pair of cells of 0.2.

The membrane potential V^I of each neuron i evolves according to the following dynamics (Gerstner & Kistler, 2002):

$$\tau_m^i \frac{dV(t)^i}{dt} = -V(t)^i + V_{leak}^i - R_m I(t)_{tot}^i$$

where τ_m is the membrane time constant, V_{leak} is the leak membrane potential, R_m is the membrane resistance and $I(t)_{tot}$ is the total input current. The neuron triggers a spike at time t^* if $V(t^*) \geq V_{thr}$ where V_{thr} is the threshold potential. Then, the membrane voltage is set to the reset membrane potential V_{reset} for the duration of the absolute refractory time period Δ.

We broke down the term $I(t)_{tot}$ into two distinct contributions, namely the sum of all the current-based synaptic inputs entering the $i-th$ neuron $I(t)_{syn}$ and the external current input $I(t)_{ext}$, as follows:

$$I(t)_{tot}^i = I(t)_{syn}^i + I(t)_{ext}^i = \sum_{j \in E} J^{j \to i}\, \mathrm{S(t)}_{\mathrm{AMPA}}^{\mathrm{j}} + \sum_{k \in I} J^{k \to i}\, \mathrm{S(t)}_{\mathrm{GABA}}^{\mathrm{k}} + I(t)_{ext}$$

where $J^{j,k \to i}$ is the efficacy of the synapsis that connects the $j,k-th$ pre-synaptic neuron to the $i-th$ post-synaptic neuron. $\mathrm{S(t)}_{\mathrm{syn}}^{\mathrm{j,k}}$ represents the synaptic dynamics which primarily depends on the synapsis type $E{:}\,AMPA, I{:}\,GABA$ (Brunel & Wang, 2003; Cavallari et al., 2014). Every time a $j,k-th$ presynaptic neuron fires at time t^*, $\mathrm{S(t)}_{\mathrm{syn}}^{\mathrm{j,k}}$ is increased by an amount described by a delayed difference of exponentials, as follows:

$$\mathrm{S(t)}_{\mathrm{syn}}^{\mathrm{j,k}} = \mathrm{S(t)}_{\mathrm{syn}}^{\mathrm{j,k}} + \Delta S(t),$$

where is defined as $\Delta S(t) = \frac{\tau_m}{\tau_d - \tau_r}\left(e^{-\frac{t-t^*-d}{\tau_d}} - e^{-\frac{t-t^*-d}{\tau_r}}\right)$, in which τ_r is the rise time, τ_d is the decay time and d is the latency of the synapsis . We defined γ-source activity as the proxy of the local field potential computed by summing the absolute values of the excitatory and inhibitory currents entering the E population (Mazzoni et al., 2015).

2. External input into the neural source model

Both populations (excitatory and inhibitory units) receive a time-varying stochastic external current input $I(t)_{ext}$ that represents the background activity from external (e.g., thalamocortical) afferents, with I neurons receiving more efficacious synapses than E neurons (Supplementary Table 1). The external input was implemented as a series of Poissonian inputs to excitatory synapses with similar kinetics to the recurrent AMPA synapses, but with different efficacy (Supplementary Table 1). These synapses were excited by independent realizations of the same Poissonian process with time-varying input rate $\nu_{ext}(t)$, and therefore, contributing to the single-neuron variability. The $\nu_{ext}(t)$ was composed of the superposition of the signal term $\nu_{signal}(t)$ and the noise term $\zeta(t)$ as follows:

$$\nu_{ext}(t) = \lfloor \nu_{signal}(t) + \zeta(\mathrm{t}) \rfloor_+$$

where $\lfloor\cdot\rfloor_+$ is the positive part operator.

We modelled the noise component of the input rate $\zeta(t)$ as a zero-mean Ornstein-Uhlenbeck (OU) process, as follows:

$$\tau_\zeta \frac{\mathrm{d}\,\zeta(t)}{\mathrm{dt}} = -\zeta(t) + \sigma_\zeta\left(\sqrt{2\tau_\zeta}\right)\eta(\mathrm{t}),$$

where $\eta(t)$ is the realization of a Gaussian white noise and σ_ζ, τ_ζ are the standard deviation and the time constant of the OU process, respectively. τ_ζ was set to have a knee in the OU power spectrum at 10 Hz (Miller et al., 2009).

3. Essential dynamics properties of the source activity

We validated essential properties - which have been already extensively investigated elsewhere (Brunel & Wang, 2003; Mazzoni et al., 2008)- of the neural dynamics network with different expressions for $\nu_{signal}(t)$: sustained signals, periodically modulated signals and signals modulated with a Gaussian profile (Supplementary Fig. 2).

First, we modeled external sources with sustained rate with amplitude varying between 2 and 20 spikes/ms. The reverberance of the E-I recurrent connections favored the emergence of reliable broadband γ-oscillations in the [60-150] Hz range (Supplementary Fig. 2). These γ-oscillations encoded the sustained signal increasing the power as the amplitude of the signal increases. It is noteworthy that this frequency range is compatible with the high-gamma speech-related spectral modulation shown in the literature (Anumanchipalli et al., 2019; Chrabaszcz et al., 2021; Proix et al., 2022).

Second, we modeled external inputs with a periodically modulated firing rate, with a varying modulation frequency between 5 and 20 Hz. The source entrained the signal fluctuations generating phase-locked oscillations at the same frequency of the periodic modulation of external input. When a superposition of the sustained and periodic signal was fed into the source, we observed the emergence of two spectral information channels: γ-oscillations that track the mean rate of the signal and low-frequency oscillations entrained by the slow time-scale component in the signal. Interestingly, the phase of the low-frequency oscillations was strongly coupled with the amplitude of γ-oscillations with surges of γ-power close to the peak of the low-frequency oscillation ($\sim \pi/8$ phase) (Supplementary Fig. 3)

Finally, to reproduce the temporal pattern of the γ-source activity, e.g., event-locked transient synchronization, we fed gaussian-modulated input rates of external signals in the model (Supplementary Fig. 1,9). The time-activation parameters of the gaussian signal (i.e., fullwidth at half maximum and mean) regulated the duration and temporal focality of the broadband γ-source synchronization (Supplementary Fig. 9).

4. Linear mixing of neural and audio sources

We simulated 99 neural γ-sources and one audio source $\mathcal{S}_a$ for each simulation. Different sources were fed by different realizations of the $\nu_{signal}(t)$ which expression depends on the simulation scenario (see below and Supplementary Table 1). Therefore, most of the variability at the source-level owes to the stochasticity in $\nu_{signal}(t)$.

In simulation settings we assumed that:

1. One source owes the major contribution to the acoustic-induced artifact ($m = 1$).
2. The audio signal $\boldsymbol{z} = z(t)$ used during the denoising pipeline is a good approximation of the artifact source $\left(\mathcal{S}_a(t) \approx z(t)\right)$, i.e., the transfer function from audio signal to speech artifact is the identity.
3. The mixing matrix A is square.

70% of γ-sources were fed by non-null $\nu_{signal}(t)$ and, as such was considered as "active". To impose the artifact-to-physiological gamma ratio (AGR), we adjusted the scale of the neural γ-sources by applying a correction factor κ, such that:

$$\kappa = \frac{\gamma_a}{\gamma_n} 10^{-AGR/10},$$

where γ_a and γ_n are the power of the audio source and neural sources in the [70-180] Hz frequency range, respectively.

We projected the neural γ-sources and the artifact source into the amplifier space $\boldsymbol{X}$ applying a random mixing matrix A. We included inter-trial variability sampling A at each simulation. By imposing the non-singularity of A, we avoided ill-posed inversion problems.

Ground truth data $\boldsymbol{X}_{gt}$, i.e., noiseless data, were generated by removing the audio source in the mixing operation.

5. Computational simulation settings

The simulations were performed using a finite difference integration scheme based on the 2nd-order Runge-Kutta algorithm with discretization time step $\Delta t = 0.05\ ms$. to update the neural dynamics. Simulation duration varied between 1 s and 10 s. We did not include the first 200 ms of each simulation in the analysis to avoid transitory dynamics in the network. We fixed the seeds of the random generator number for sake of the reproducibility across different simulation sessions.

Supplementary Tables

Table S1: LIF network parameters and external input definitions for different in-silico scenarios. List of parameters used for in-silico brain sources. $N(\mu, \sigma)$ indicates the gaussian distribution with mean μ and standard deviation σ. $U(a, b)$ defines the continuous uniform distribution with support $[a, b]$.

**Network structure**		
Number of neurons	N_E	4000
	N_I	1000
Connection probability	p	0.2
**Neuron dynamics**		
Discretization step	Δt	0.05 ms
Leak membrane potential	V_{leak}^E	-70 mV
	V_{leak}^I	-70 mV
Threshold membrane potential	V_{thr}^E	-52 mV
	V_{thr}^I	-52 mV
Reset membrane potential	V_{reset}^E	-59 mV
	V_{reset}^I	-59 mV
Absolute refractory period	Δ^E	2 ms
	Δ^I	1 ms
Membrane resistance	R_m^E	0.04 $G\Omega$
	R_m^I	0.05 $G\Omega$
Membrane time constant	τ_m^E	20 ms
	τ_m^I	10 ms
**Synapses**		
Synaptic efficacy	$J^{E\to E}$	-10.5 pA
	$J^{I\to E}$	42.5 pA
	$J^{ext\to E}$	-13.75 pA
	$J^{I\to I}$	54 pA
	$J^{E\to I}$	-14 pA
	$J^{ext\to I}$	-19 pA
Synaptic delay	$d^{E\to E}$	1 ms
	$d^{I\to E}$	1 ms
	$d^{ext\to E}$	1 ms
	$d^{I\to I}$	1 ms
	$d^{E\to I}$	1 ms
	$d^{ext\to I}$	1 ms
Synaptic rise constant	$\tau_r^{E\to E}$	0.40 ms
	$\tau_r^{I\to E}$	0.25 ms
	$\tau_r^{ext\to E}$	0.40 ms
	$\tau_r^{I\to I}$	0.25 ms
	$\tau_r^{E\to I}$	0.20 ms
	$\tau_r^{ext\to I}$	0.20 ms
Synaptic decay constant	$\tau_d^{E\to E}$	2 ms
	$\tau_d^{I\to E}$	5 ms
	$\tau_d^{ext\to E}$	2 ms
	$\tau_d^{I\to I}$	5 ms

	$\tau_d^{E\to I}$	1 ms
	$\tau_d^{ext\to I}$	1 ms
**External input $I_{ext}(t)$ in Toy examples: SAS, CAS, MCAS** $A_s + A_p \sin(2\pi f_p t + \varphi_p) + \zeta(t)$		
Static amplitude	A_s	$U(12{,}16)$ $spike/(ms * cell)$
Periodic amplitude	A_p	$N\left(0, \frac{16}{3}\right) spike/(ms * cell)$
Frequency oscillation	f_p	$N(10{,}1.5)$ Hz
Phase oscillation	φ_p	$U(-\pi, \pi)$
UO constant time	τ_ζ	0.16 s
UO standard deviation	σ_ζ	4 spikes/ms
**External input $I_{ext}(t)$ in Realistic simulations: RAS** $\mathrm{Ae}^{-\frac{(t-\mu)^2}{\left(2FWHM/2.355\right)^2}} + \zeta(t)$		
Amplitude modulation	A	$U(10{,}14)$ $spike/(ms * cell)$
Peak modulation time	μ	$N(5,\ 0.05)$s
Full width at half maximum	$FWHM$	$N(1.2,\ 0.15)$s
UO constant time	τ_ξ	0.16 s
Uo standard deviation	σ_ζ	4 spikes/ms

Table S2: Audio definitions for the different toy examples: SAS, CAS, MCAS. List of parameters used for in-silico audio sources. $\sigma\eta(t)$ indicates the white noise with mean 0 and standard deviation σ.

**Toy examples**	**Sinusoidal audio scenario (SAS):** $A_0 sin(2\pi F_0 t + \varphi_0) + \eta(t)$		
	Amplitude audio source	A_0	$15 \cdot 10^3$
	Fundamental frequency	F_0	120 Hz
	Phase audio source	φ_0	$\pi/3$
	**Colored noise audio scenario (CAS):** $\sigma\eta(t)$		
	Standard deviation white noise	σ	$15 \cdot 10^{15}$
	Center frequency filter	F_0	120 Hz
	Bandwidth filter	ΔF	5 Hz
	Order filter	b	25
	**Modulated colored noise audio scenarios (MCAS):** $\sigma\eta(t) \rightarrow$ BP filter $\rightarrow M(t) \rightarrow$		
	Standard deviation white noise	σ	$15 \cdot 10^{15}$
	Center frequency filter	F_0	120 Hz
	Bandwidth filter	ΔF	5 Hz
	Order filter	b	25
	# bumps		3
	Duration bump		0.5 s

Supplementary Figures

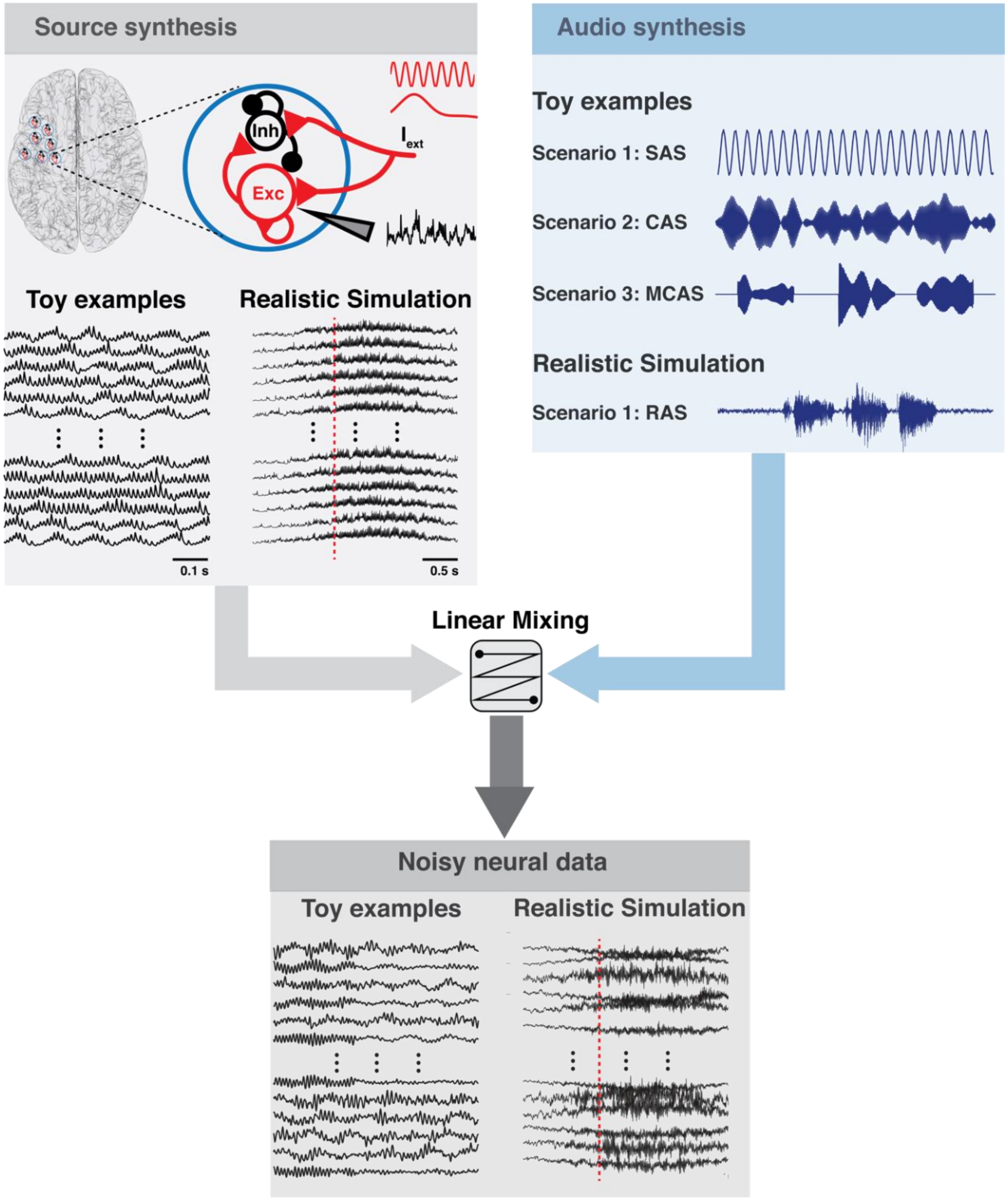

Supplementary Fig. 1 | Schematic of the pipeline for the simulation scenarios. Brain sources are simulated using a sparse (sparseness; p = 0.2) LIF network of excitatory (N= 4000, red line) and inhibitory neurons (N = 1000, black line). Both populations receive recurrent activity and external excitatory inputs I_{ext}. I_{ext} is a Poissonian process with time-varying input rate $\nu_{ext}(t)$. The size of the synaptic connection (inhibitory: circle and excitatory: triangle) depicts the synaptic efficacy. LFPs are estimated using a simple computational proxy which neglects the direct contribution of the inhibitory population (refer to for details). Simulation of neural data affected by the vibration artifact are obtained by linearly mixing brain sources and audio signal by the application of a mixing matrix. We simulated different scenarios according to the type of audio signal and the $\nu_{ext}(t)$ expression (refer to Methods for details). The vertical red dashed line depicts the onset of the speech production event. (SAS: sinusoidal audio scenario, CAS: colored noise audio scenario, MCAS: modulated colored noise scenario, RAS: recorded audio scenario)

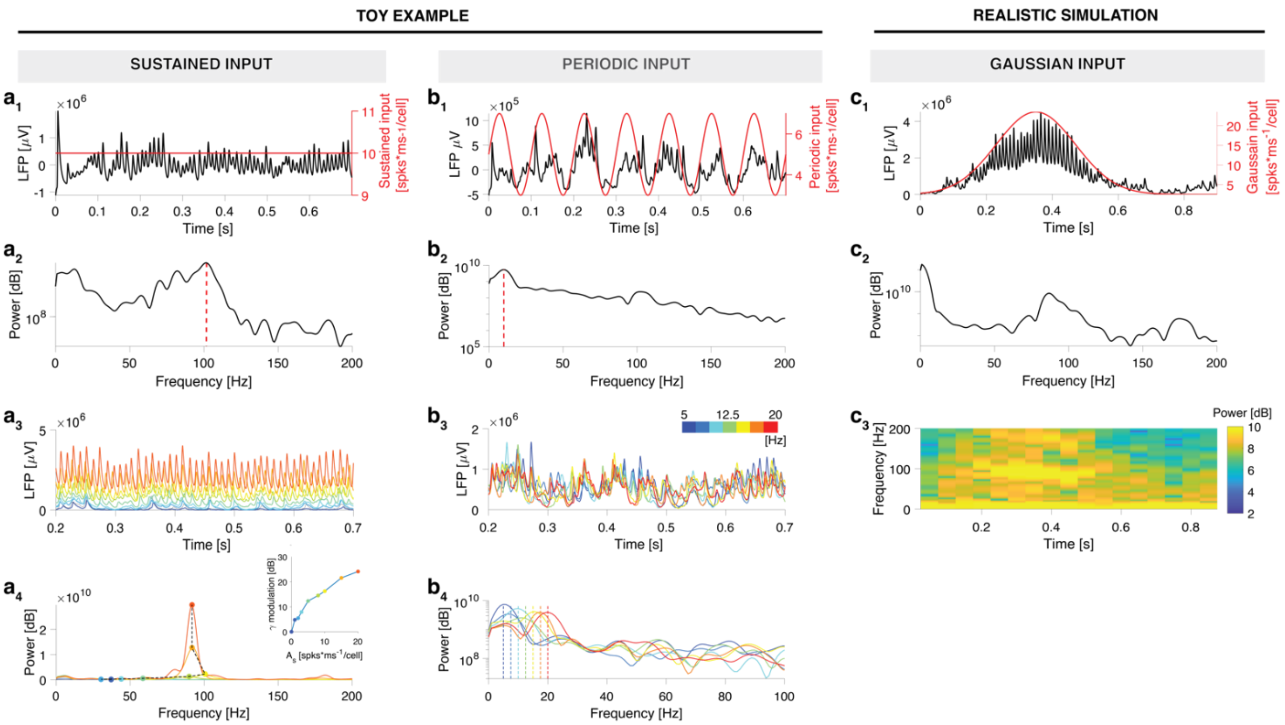

Supplementary Fig. 2 | Source activity reflects temporal properties of the network input $\nu_{signal}(t)$. a_1- a_2, Temporal and spectral source activity during the injection of a sustained input at 10 spikes/(ms*cell) (red line). Strong γ-oscillations are visible in the time domain with a peak at ~100 Hz (vertical red dashed line). **a_3- a_4**, gamma-oscillations entrainment can be tweaked by sweeping the intensity of the sustained input, as depicted by the color code. **b_1- b_2**, Temporal and spectral source activity during the injection of a periodically modulated input rate at 10 Hz (red line). Source activity tracks the frequency of the periodic input, revealing a peak at 10 Hz (vertical red dashed line). **b_3- b_4**, High fidelity between the frequency of the periodic input (vertical dashed line) and the peak of the oscillatory source activity, as depicted by the color code. **c_1- c_3** Time-frequency representation of the source activity during the injection of a gaussian-modulated input. Transient gamma-oscillations emerge between 0.2 s and 0.5 s.

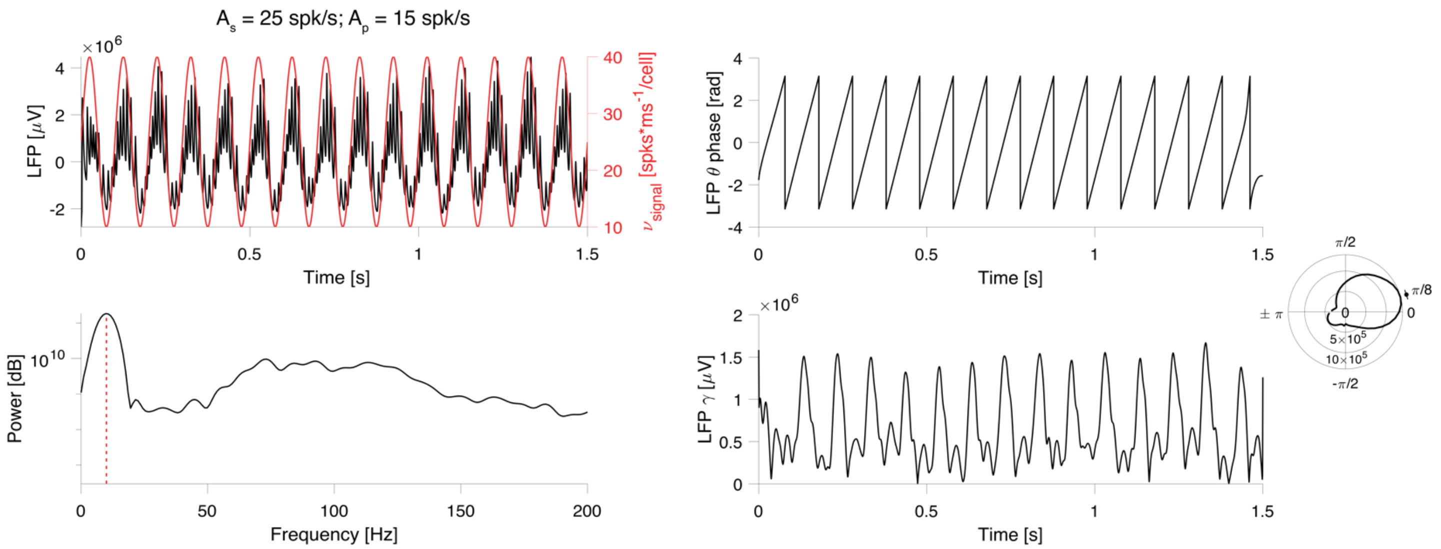

Supplementary Fig. 3 | Neural source activity reproduces physiological phase-amplitude coupling. Temporal and spectral source activity during the injection of a periodic input at 10 Hz (red line). Source activity tracks the oscillation frequency of the period input (vertical red dashed line). When dissecting spectral components of the source activity, phase of the low-frequency oscillations was strongly coupled with the amplitude of γ-oscillations with surges of γ-power close to the peak of the low-frequency oscillation ($\sim \pi/8$ phase). Inset plot shows γ-power w.r.t the phase of the low frequency oscillation.

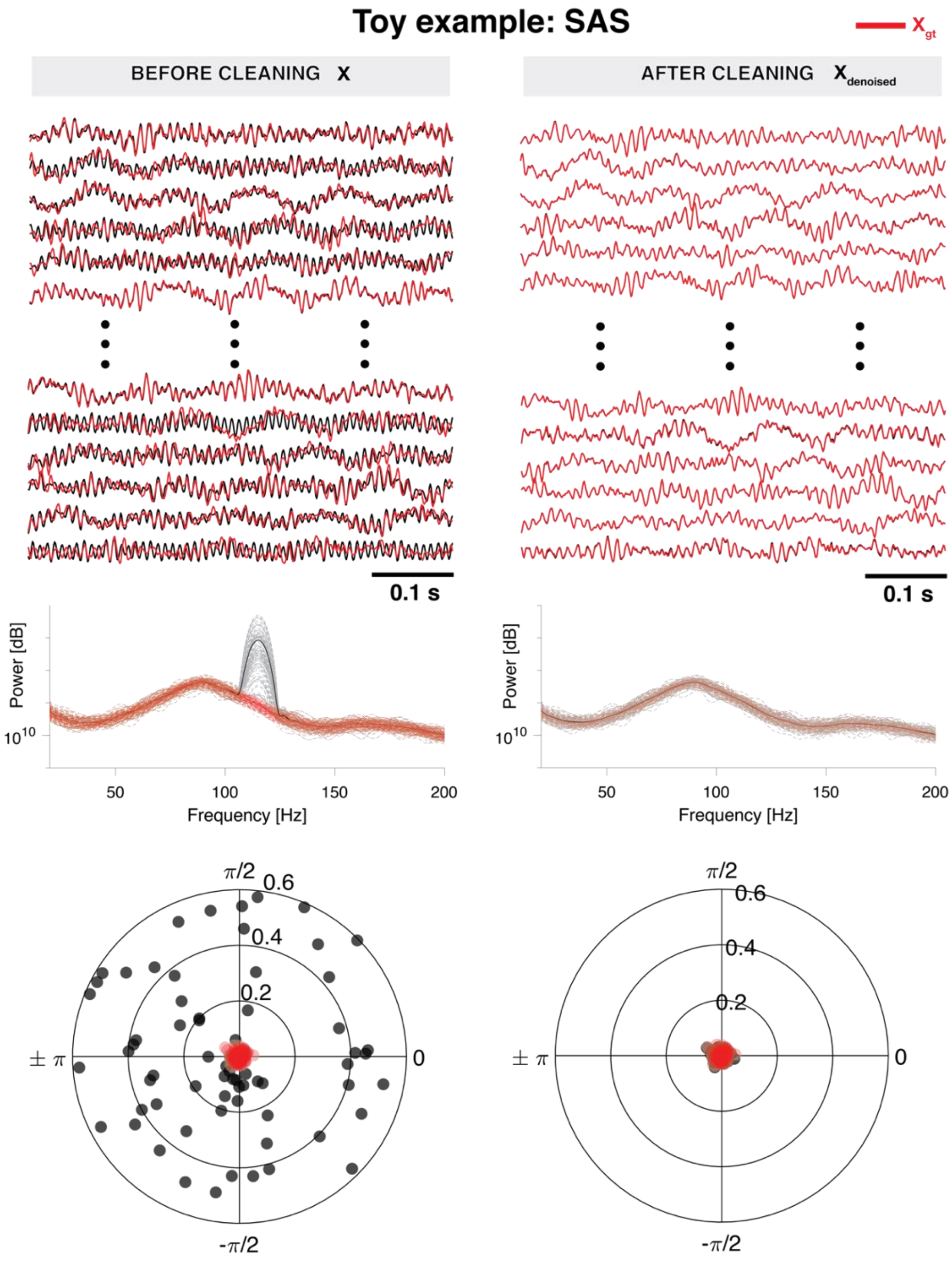

Supplementary Fig. 4 | PCD perfectly removes the audio source in the toy example SAS. Noisy neural sources **(**$\boldsymbol{X}$, black line, left) are obtained by linear mixing of ground-truth neural sources ($\boldsymbol{X_{gt}}$, red line) with a sinusoidal audio source ($F_0 = 120\ Hz$). $\boldsymbol{X}$ exhibits a narrowband component around the fundamental frequency F_0 as well as phase locking with the audio source. After cleaning, $\boldsymbol{X_{denoised}}$ (black line, right) resembles $\boldsymbol{X_{gt}}$ in the time and frequency domain. Finally, $\boldsymbol{X_{denoised}}$ is uncoupled with the audio source.

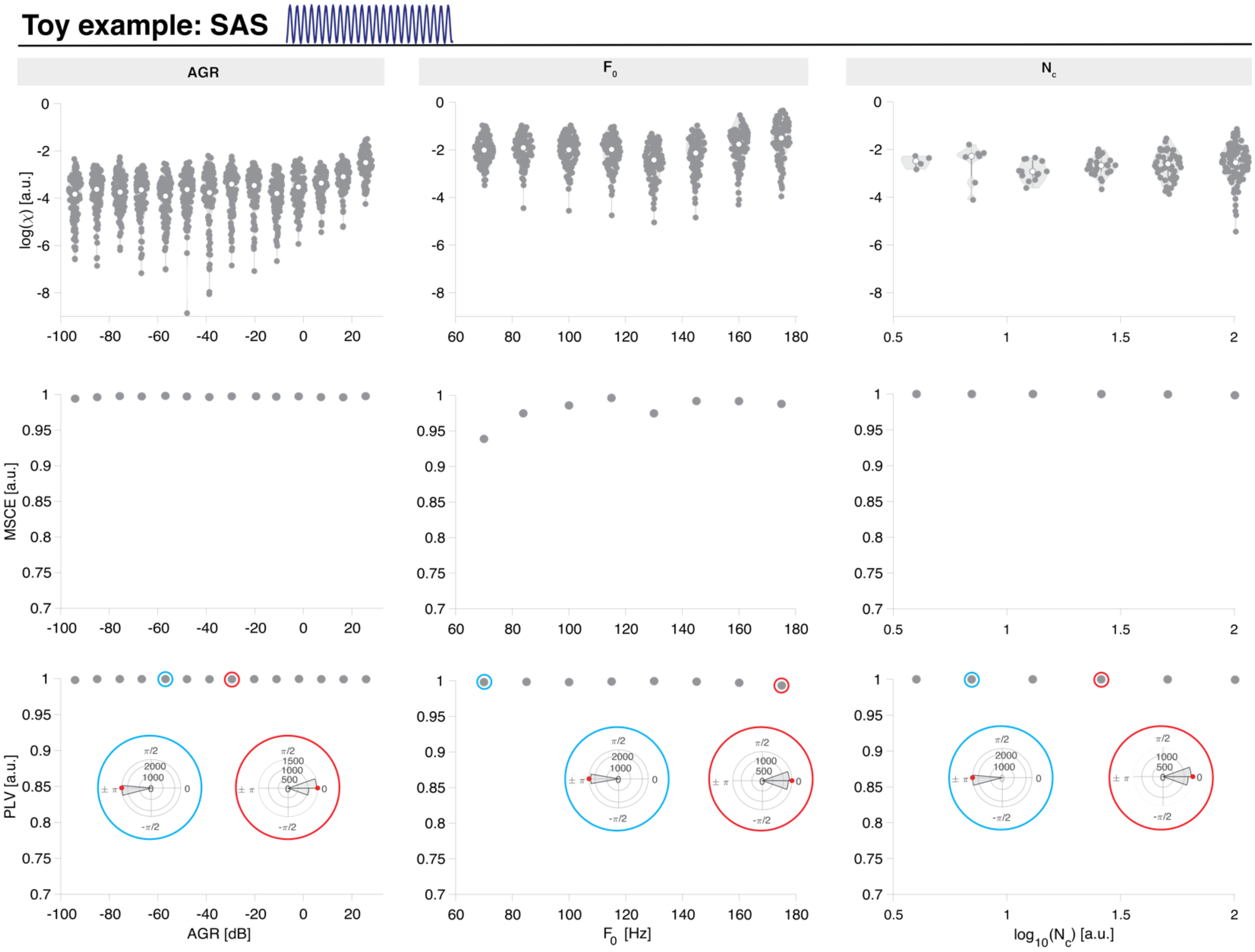

Supplementary Fig. 5 | Performances of PCD pipeline in the toy example SAS across a range of AGR, $\boldsymbol{F_0}$, and $\boldsymbol{N_C}$. PCD pipeline is robust to AGR (**a**), $\boldsymbol{F_0}$ (**b**) and $\boldsymbol{N_C}$ (**c**) changes in terms of agreement between the ground-truth $\boldsymbol{X_{gt}}$ and cleaned data $\boldsymbol{X}$ (top, $\log(\chi)$) and between the artefact z(t) and the estimated artefact $z(t)_{est}$ (center-bottom, MSCE and PLV). Inset polar plots show the phase difference (z(t) vs. $z(t)_{est}$) distribution in two exemplary simulations (red: correlation, cyan: anticorrelation), as pinpointed by surrounding circles Red filled circle displays the average.

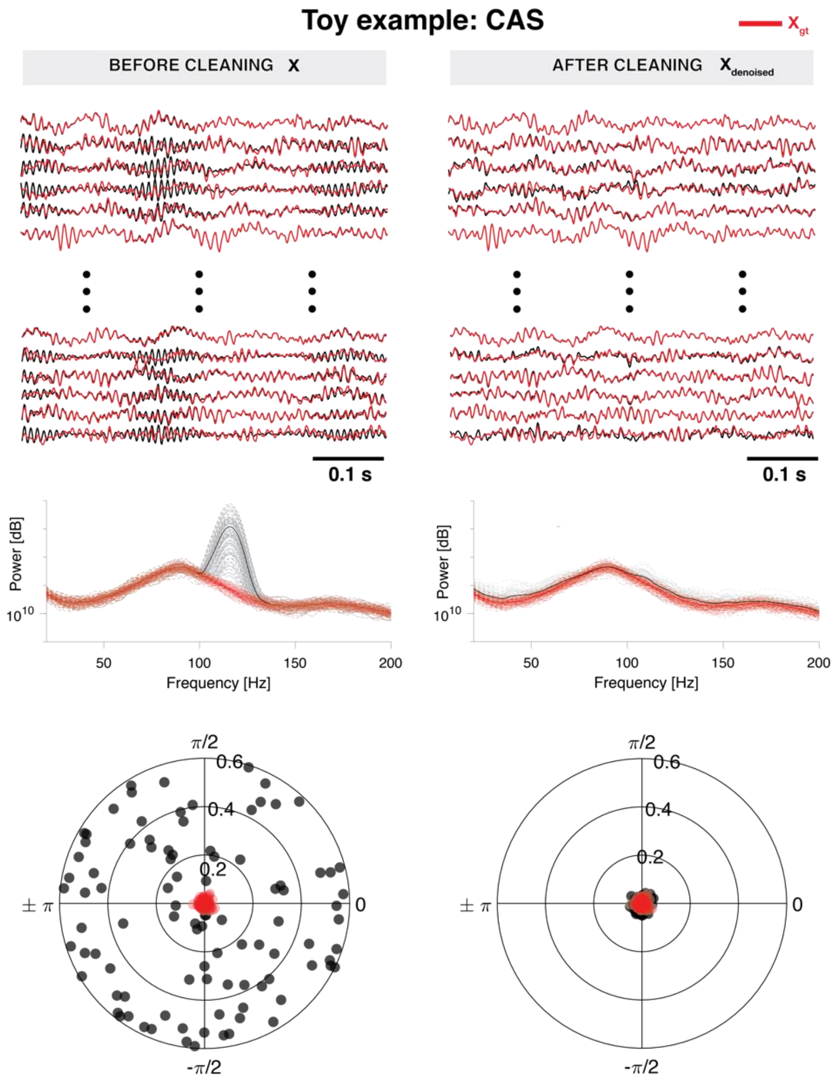

Supplementary Fig. 6 | PCD perfectly removes the audio source in the toy example CAS. 60 trials of noisy neural sources **(*X***, black line, left) are obtained by linear mixing of ground-truth neural sources ($\boldsymbol{X_{gt}}$, red line) with a colored noise audio source ($F_0 = 120\,Hz, \Delta F = 5\,Hz$, duration bump = 0.5 s).***X*** exhibits a narrowband component around the fundamental frequency F_0 as well as phase locking with the audio source. After cleaning, $\boldsymbol{X_{denoised}}$ (black line, right) resembles $\boldsymbol{X_{gt}}$ in the time and frequency domain. Finally, $\boldsymbol{X_{denoised}}$ is uncoupled with the audio source. Performance metrics ($\log(\chi)$, MSCE and PLV) distributions are displayed.

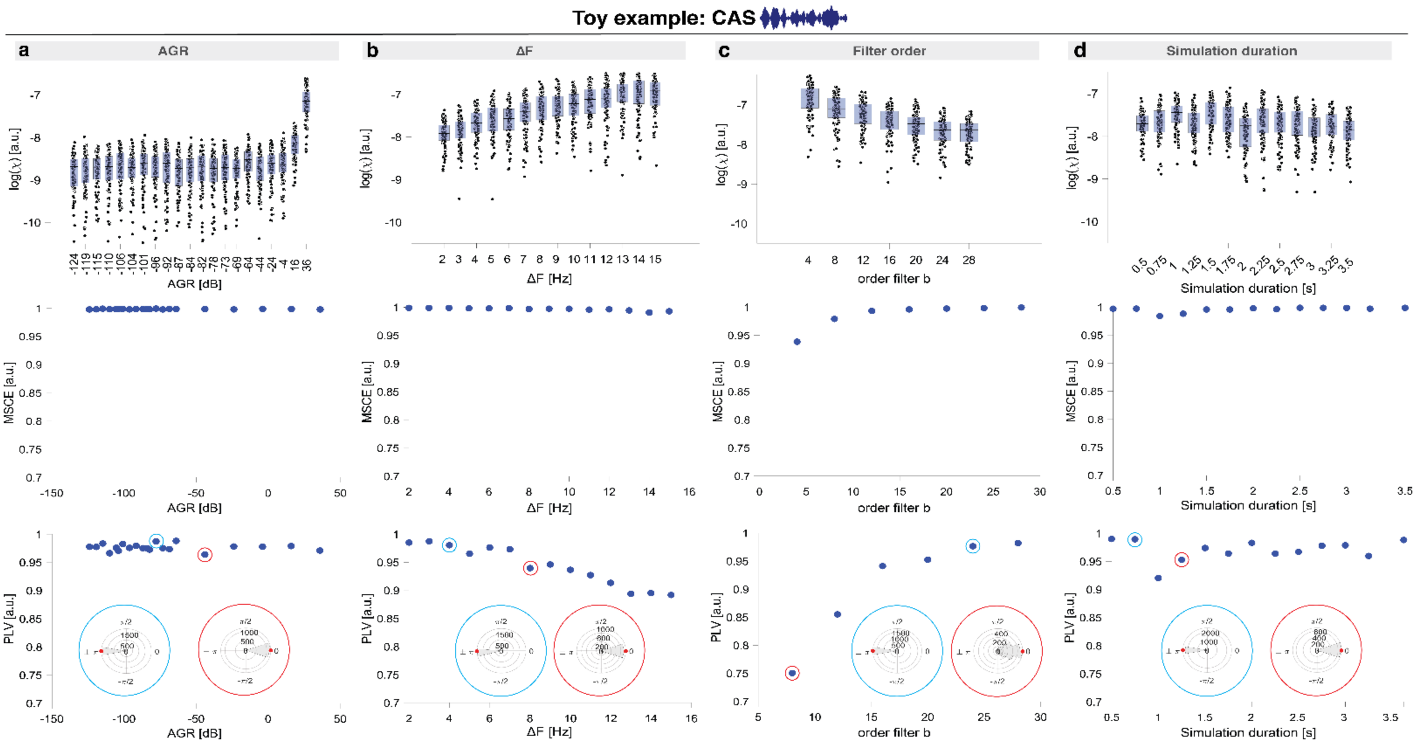

Supplementary Fig. 7 | Performances of PCD pipeline in the toy example CAS across a range of AGR, ΔF, filter order b and simulation duration. a, PCD pipeline is robust to artifact-to-physiological gamma ratio (AGR) changes in terms of agreement between the ground-truth $\boldsymbol{X}_{gt}$ and cleaned data $\boldsymbol{X}$ (top, $log(\chi)$) and between the artifact $z(t)$ and the estimated artifact $z(t)_{est}$ (center-bottom, MSCE and PLV). **b-c, Broad SAFB significantly reduces PCD performances.** PCD pipeline is more accurate to remove narrowband artifacts, as suggested by the drop in performances when either the artifact frequency peak is too large (ΔF) or not well defined (order filter b) (Section 2.2.1.1, CAS artifact definition). **d,** Duration of the simulation does not significantly impact PCD performances. Inset polar plots show the phase difference ($z(t)$ vs. $z(t)_{est}$) distribution in two exemplary simulations (red: correlation, cyan: anticorrelation), as pinpointed by surrounding circles Red filled circle displays the average.

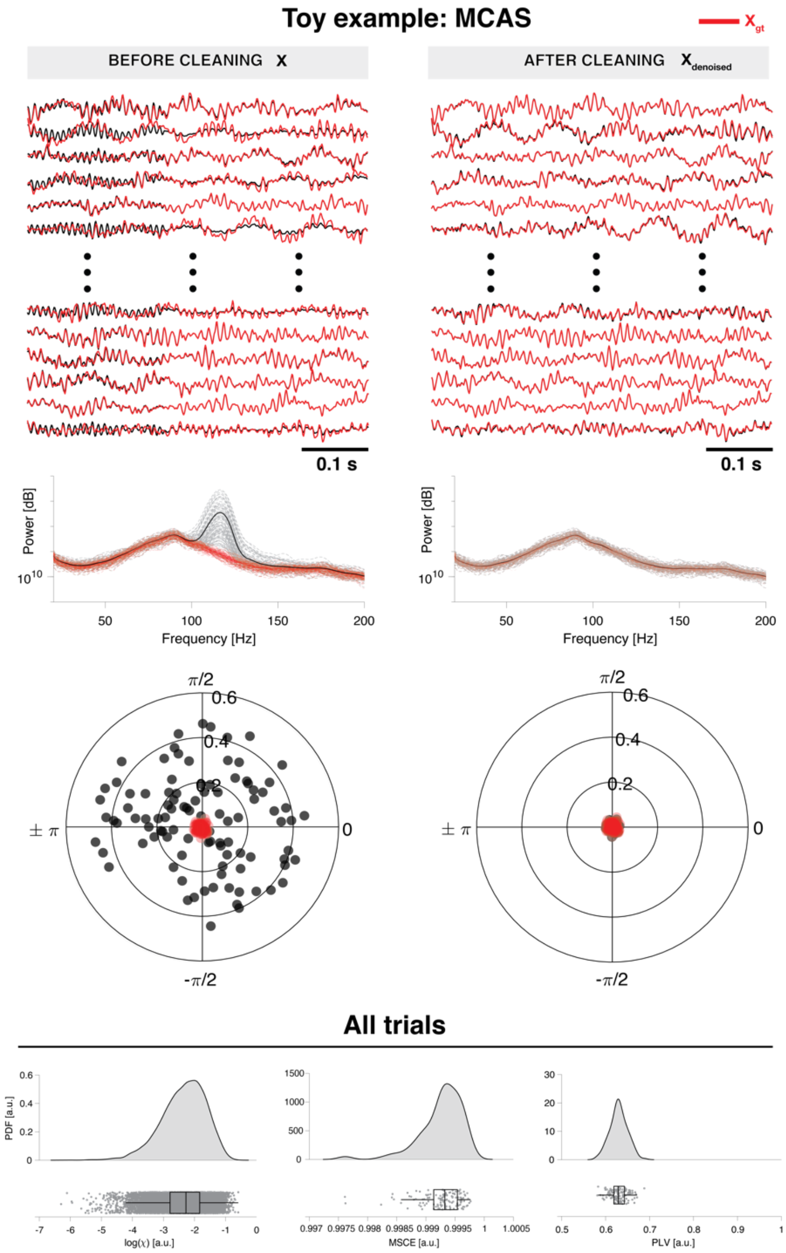

Supplementary Fig. 8 | PCD perfectly removes the audio source in the toy example MCAS. Noisy neural sources (**X**, black line, left) are obtained by linear mixing of ground-truth neural sources (**X_{gt}**, red line) with a modulated colored noise audio source ($F_0 = 120\ Hz, \Delta F = 5\ Hz$). **$X$** exhibits a narrowband component around the fundamental frequency F_0 as well as phase locking with the audio source. After cleaning, **$X_{denoised}$** (black line, right) resembles **X_{gt}** in the time and frequency domain. Finally, **$X_{denoised}$** is uncoupled with the audio source.

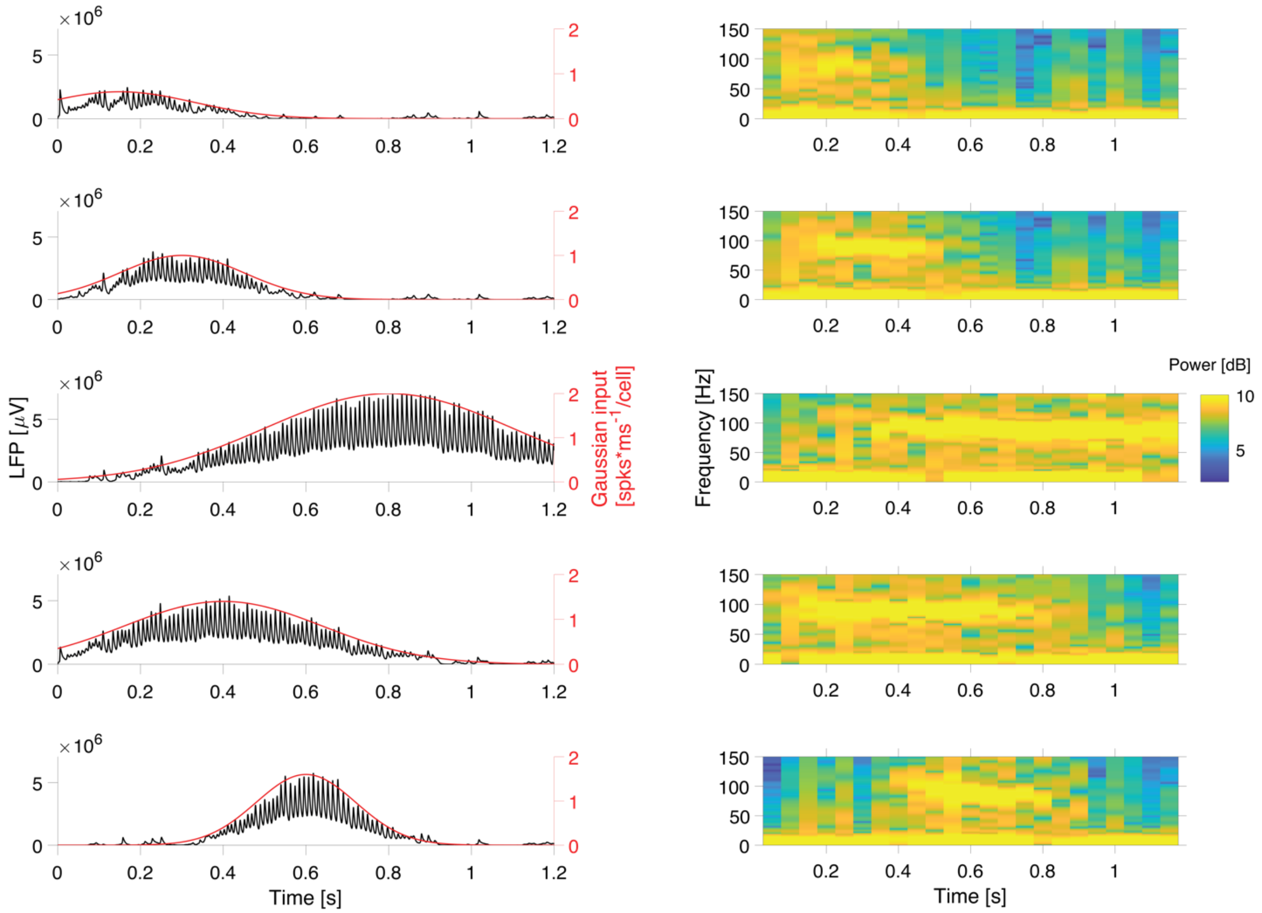

Supplementary Fig. 9 | Gaussian input in the source generates different patterns of event-locked transient activity. Exemplary time-frequency representation of the neural source fed by a gaussian input (red line). The duration and the temporal focality of the broadband γ-power activity can be tweaked by manipulating the mean (time of peak modulation) and the fullwidth at half maximum (modulation duration) of the gaussian function.

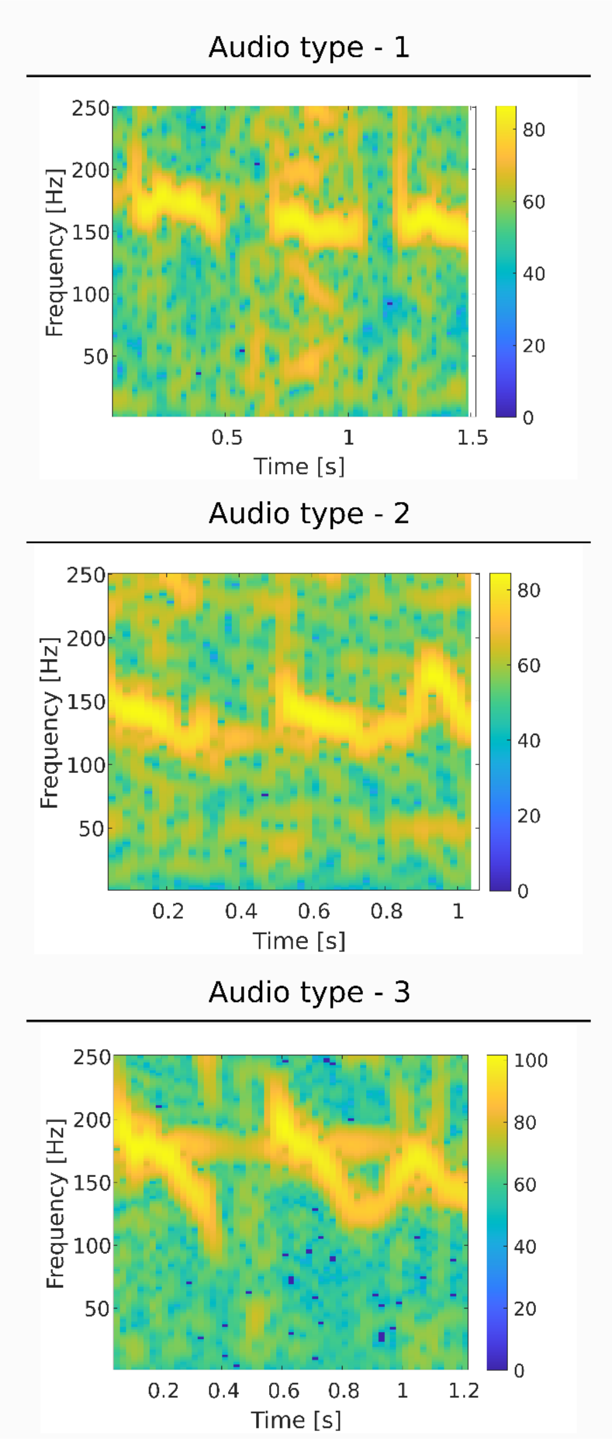

Supplementary Fig. 10 | Spectrogram of the recorded audio used for the realistic scenario. Spectrograms of different pitch patterns of utterances from three different participants.

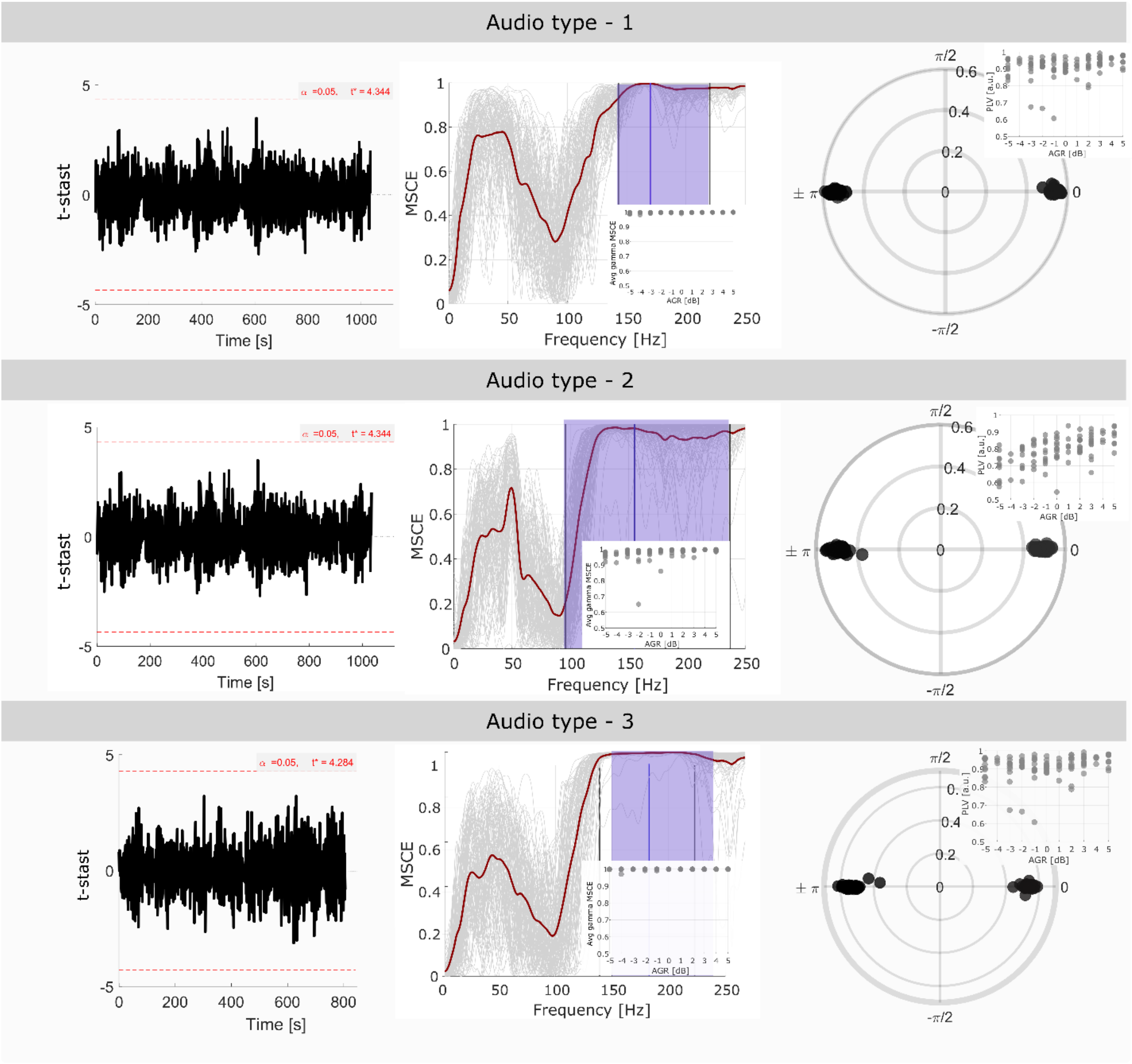

Supplementary Fig. 11 | PCD perfectly retrieves the audio source in realistic simulated scenarios. For every type of audio used during the recorded audio scenario (RAS) the PCD artifact estimation capacity is evaluated across trials in the time, frequency and phase domains. First column: one-dimensional statistical parametric mapping (SPM) used to evaluate statistical similarities at each sample point between the true and estimated source for each trial. No significant differences (t-stast values < t*) were found at any time point, indicating that across trials the true and estimated artifact source are identical from a statistical viewpoint. Second column: The magnitude-squared coherence estimate (MSCE) was computed between the true and the estimated artifact source. Gray lines represent individual trials. Mean MSCE across trials is denoted by the dark red line. The mean F0 across trials is shown as the blue vertical line, while the violet band indicates the possible range of speech artifact frequency band (SAFB) variations. The mean MSCE value in the SAFB was always above 0.97, regardless of the AGR of the trials. Third column: Phase differences between the true and the estimated artifact source. Each dot represents a trial phase difference. Estimated sources were either in phase or anti-phase relationship to the true source. Figure on the right corner aggregates the phase-locking value (PVL) across simulated trials at different AGR.

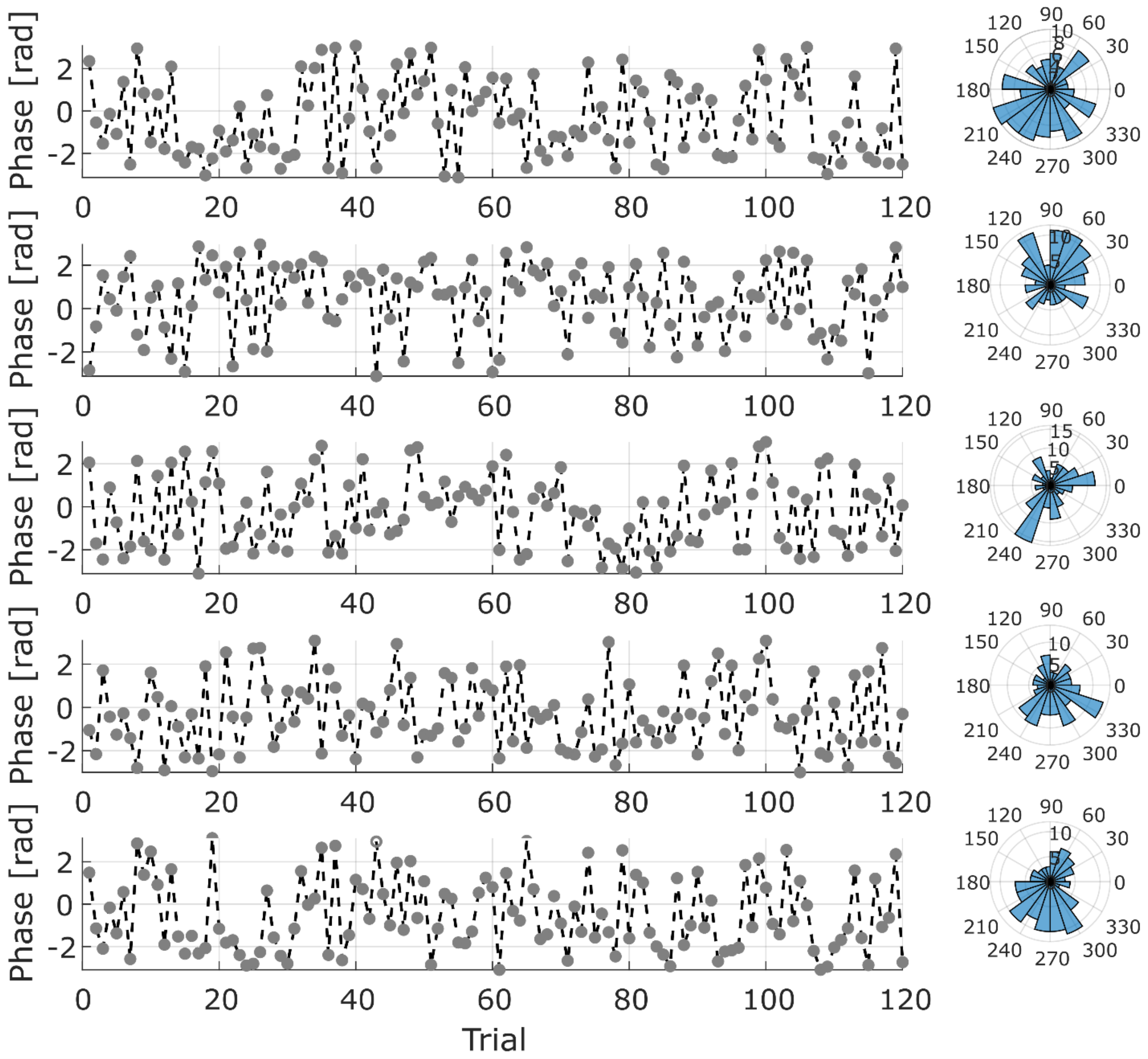

Supplementary Fig. 12 | Artifact phase relationship varies across trials. For a given participant and 5 random selected channels, the mean phase relationship between the recorded audio and each channel is shown for each trial. Figures on the right aggregate the distribution of the mean phase values across trials.

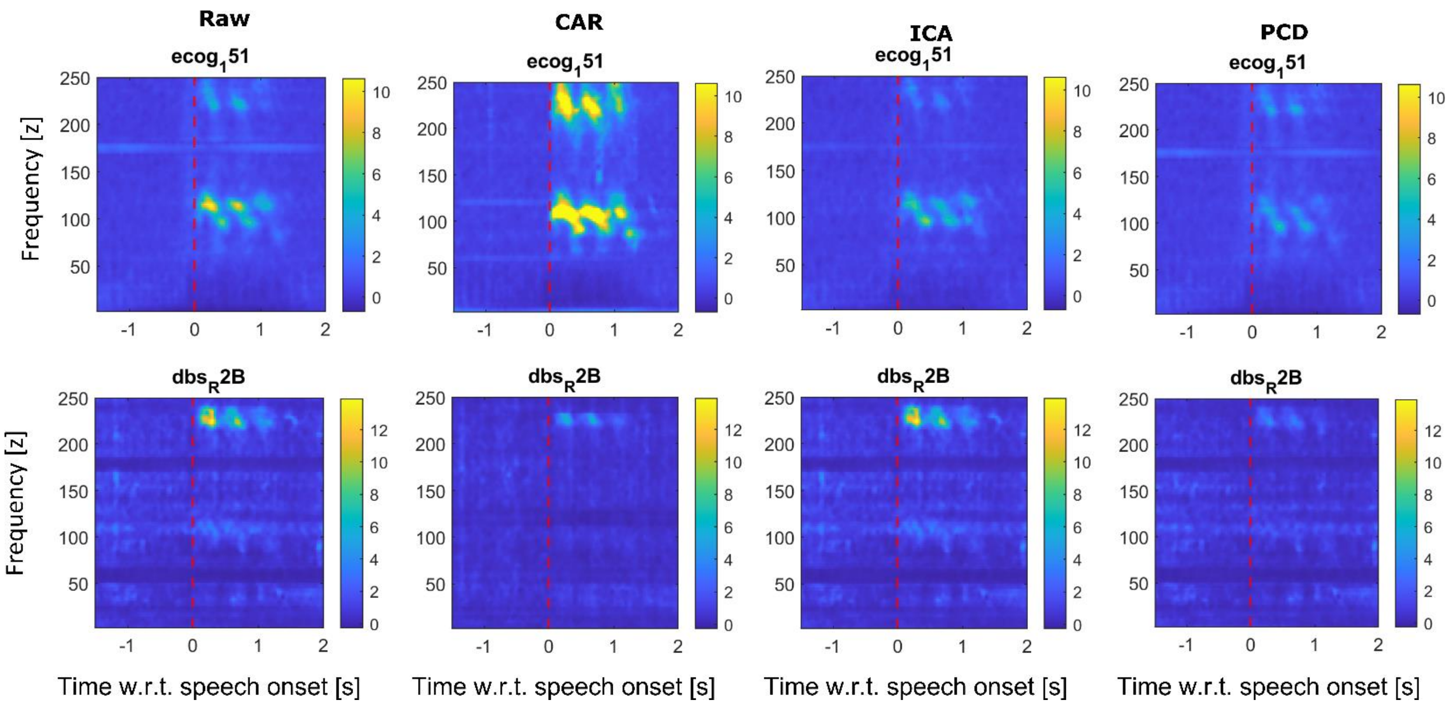

Supplementary Fig. 13. Methods performance in artifactual electrodes at the first harmonic of F0. Time-frequency plots for raw signals and effect of applying each denoising method in electrode with speech artifact at the first harmonic of F0**.** (top) ECoG electrode, (bottom) DBS electrode**.** Time is relative to speech onset

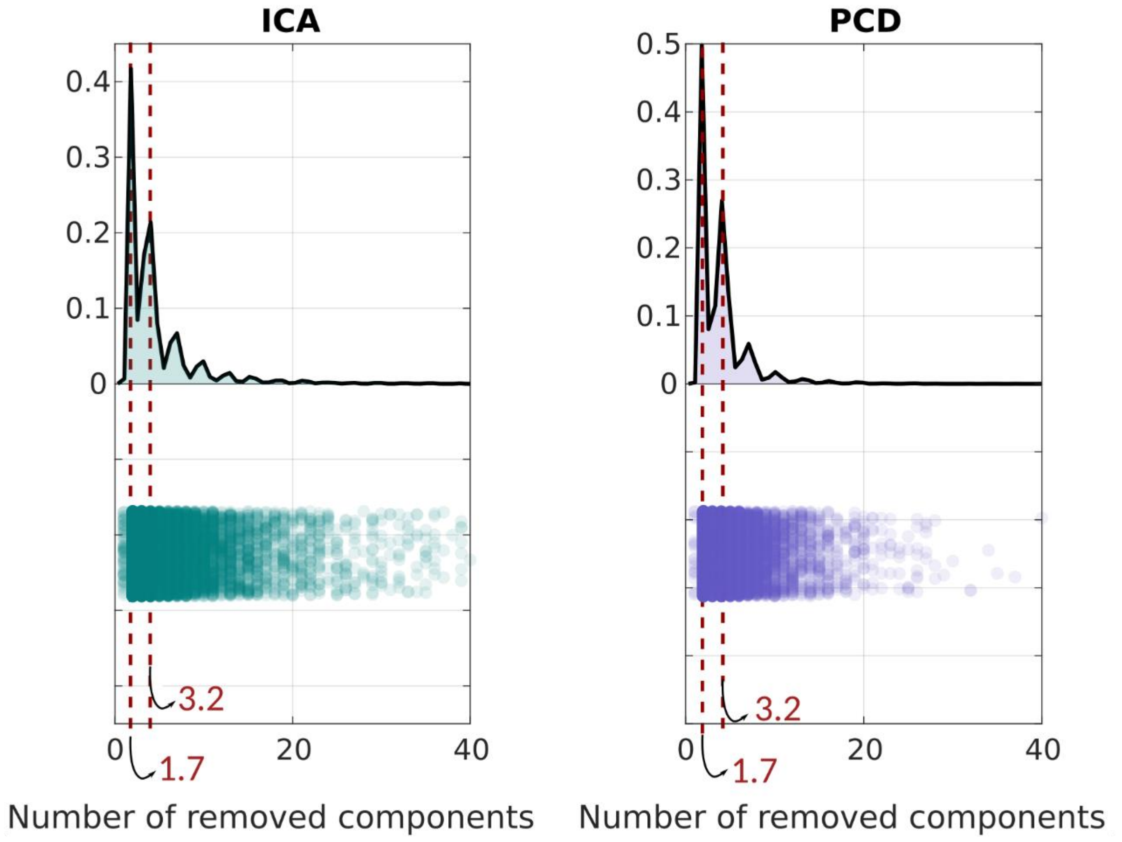

Supplementary Fig. 14 | Number of removed components by ICA and PCD. For each subject at each recording session, the number of removed component as automatically selected by ICA and PCD is drawn. Both methods in most of the cases removed around 1 to 4 components.

Supplementary References

Anumanchipalli, G. K., Chartier, J., & Chang, E. F. (2019). Speech synthesis from neural decoding of spoken sentences. *Nature*, *568*(7753), 493–498. htI tps://doi.org/10.1038/s41586-019-1119-1

Brunel, N., & Wang, X.-J. (2003). What Determines the Frequency of Fast Network Oscillations With Irregular Neural Discharges? I. Synaptic Dynamics and Excitation-Inhibition Balance. *Journal of Neurophysiology*, *90*(1), 415–430. https://doi.org/10.1152/jn.01095.2002

Buzsáki, G., & Wang, X.-J. (2012). Mechanisms of Gamma Oscillations. *Annual Review of Neuroscience*, *35*(1), 203–225. https://doi.org/10.1146/annurev-neuro-062111-150444

Cavallari, S., Panzeri, S., & Mazzoni, A. (2014). Comparison of the dynamics of neural interactions between current-based and conductance-based integrate-and-fire recurrent networks. *Frontiers in Neural Circuits*, *8*. https://doi.org/10.3389/fncir.2014.00012

Chrabaszcz, A., Wang, D., Lipski, W. J., Bush, A., Crammond, D. J., Shaiman, S., Dickey, M. W., Holt, L. L., Turner, R. S., Fiez, J. A., & Richardson, R. M. (2021). Simultaneously recorded subthalamic and cortical LFPs reveal different lexicality effects during reading aloud. *Journal of Neurolinguistics*, *60*, 101019. https://doi.org/10.1016/j.jneuroling.2021.101019

Gerstner, W., & Kistler, W. M. (2002). *Spiking Neuron Models: Single Neurons, Populations, Plasticity* (1st ed.). Cambridge University Press. https://doi.org/10.1017/CBO9780511815706

Mazzoni, A., Lindén, H., Cuntz, H., Lansner, A., Panzeri, S., & Einevoll, G. T. (2015). Computing the Local Field Potential (LFP) from Integrate-and-Fire Network Models. *PLOS Computational Biology*, *11*(12), e1004584. https://doi.org/10.1371/journal.pcbi.1004584

Mazzoni, A., Panzeri, S., Logothetis, N. K., & Brunel, N. (2008). Encoding of Naturalistic Stimuli by Local Field Potential Spectra in Networks of Excitatory and Inhibitory Neurons. *PLoS Computational Biology*, *4*(12), e1000239. https://doi.org/10.1371/journal.pcbi.1000239

Meneghetti, N., Cerri, C., Tantillo, E., Vannini, E., Caleo, M., & Mazzoni, A. (2021). Narrow and Broad γ Bands Process Complementary Visual Information in Mouse Primary Visual Cortex. *Eneuro*, *8*(6), ENEURO.0106-21.2021. https://doi.org/10.1523/ENEURO.0106-21.2021

Miller, K. J., Sorensen, L. B., Ojemann, J. G., & Den Nijs, M. (2009). Power-Law Scaling in the Brain Surface Electric Potential. *PLoS Computational Biology*, *5*(12), e1000609. https://doi.org/10.1371/journal.pcbi.1000609

Proix, T., Delgado Saa, J., Christen, A., Martin, S., Pasley, B. N., Knight, R. T., Tian, X., Poeppel, D., Doyle, W. K., Devinsky, O., Arnal, L. H., Mégevand, P., & Giraud, A.-L. (2022). Imagined speech can be decoded from low- and cross-frequency intracranial EEG features. *Nature Communications*, *13*(1), 48. https://doi.org/10.1038/s41467-021-27725-3
